# Emergency Hospital Admissions for Cardiovascular Causes Attributable to Air Pollution and Extreme Temperatures in Spain: Influence of Economic and Demographic Factors in a Nationwide Study

**DOI:** 10.1007/s11524-025-01006-6

**Published:** 2025-09-02

**Authors:** J. A. López-Bueno, J. Díaz, M. Iriso, R. Ruiz-Páez, M. A. Navas-Martín, C. Linares

**Affiliations:** 1 National School of Public Health, Climate Change, Health and Urban Environment Reference Unit, Carlos III Institute of Health (Instituto de Salud Carlos III/ISCIII), Avda. Monforte de Lemos 5, 28029 Madrid, Spain; 2 Madrid Complutense University, Madrid, Spain; 3 University of Alcalá, Madrid, Spain

**Keywords:** Hospital admissions, Cardiovascular diseases, Air pollution, Extreme temperatures, Demographic and socioeconomic factores

## Abstract

Using generalised linear models (GLMs) with a Poisson link, this study analysed the short-term effect of NO_2_, ozone, PM_10_ and PM_2.5_ on the daily number of emergency hospital admissions due to cardiovascular disease (CVD), acute cerebrovascular disease, acute myocardial infarction (AMI) and ischaemic heart disease (IHD). The impact of the minimum daily temperature in cold waves and maximum daily temperature in heat waves was likewise analysed. Specific dose–response functions were calculated for each province and cause of admission considered. We used random effects mixed models to analyse the relationship between the percentage of admissions in each province and the following provincial variables: income level; number of inhabitants; population percentage aged 65 years and over and percentage of women. Our results indicated that in Spain there were 10,167 (95%CI: 3.679, 16.554) emergency CVD-related annual admissions attributable to the variables considered, which accounted for 7.7% of total annual CVD-related admissions in Spain and broke down as follows: 6.9% attributable to air pollution in general, and NO_2_ and O_3_ in particular, and 0.8% attributable to extreme temperatures, especially during cold waves. A similar pattern could be observed for the specific causes. With respect to the percentage of CVD-related admissions attributable to air pollution, higher income level was a protective element and age over 65 years was a risk factor for the percentage of the population concerned. Air pollution is a risk factor for short-term emergency hospital admissions due to CVD in Spain, with the effect NO_2_ and O_3_ being especially noteworthy, in terms of all CVD-related causes and the specific causes considered. The impact of extreme temperatures is of a lower order of magnitude than that of air pollution, and the effect of the maximum daily temperature in heat waves on CVD is extremely small.

**Supplementary Information:**

The online version contains supplementary material available at 10.1007/s11524-025-01006-6.

## Introduction

Environmental air pollution is an important public health problem that affects the population of all countries. In 2019, 99% of the world population lived in places where World Health Organisation (WHO) air quality guideline levels were not observed, and around 68% of premature deaths worldwide linked to outdoor air pollution were estimated to be due to ischaemic heart diseases and cerebrovascular accidents [69]. In Europe, air pollution is one of the main environmental risks for health and is perceived to be so by the general public [18]. Indeed, the evidence shows that even low levels of exposure are associated with harmful impacts [6], such as increased morbidity and mortality due to circulatory causes [60]. In Spain, the main air pollutants that are harmful to health are nitrogen dioxide (NO_2_), tropospheric ozone (O_3_), particulate matter having a diameter of 2.5 microns or less (PM_2.5_) and particulate matter having a diameter of 10 microns or less (PM_10_) [44]. While most of the literature published on the health effects of air pollution is based on an analysis of mortality, due in the main to long-term exposure to pollutants, analysing the effect on morbidity helps to ascertain how the population’s quality of life can be improved and more rational use made of healthcare expenditure [67]. Moreover, it makes it possible to study the impact of pollution on diseases which, while not necessarily mortal, are nevertheless the cause of a great number of hospitalisations. Furthermore, studying the effect of short-term exposure to air pollution makes it possible to evaluate the immediate impact of episodic increases in pollution levels, whose effects on population health tend to become evident relatively soon. This approach is especially valuable for health policy decision-makers when drawing up plans targeted at controlling and mitigating the effects of pollution on health. Indeed, this is why 24-h values are included in both the WHO guidelines and the new EU Directive [10].

In terms of their influence on the health impact of air pollution, a number of fundamental factors must be borne in mind, including temperature and other intercorrelated meteorological variables [2]. Many studies have analysed the interaction between extreme temperature events and air pollution [25, 48, 51, 61]. It should be stressed that, despite there being ample documentation on the health impacts of O_3_, NO_2_ and PM, the results differ widely when it comes to analysing whether high temperatures influence these associations. This variability can be ascribed to the diversity of definitions used for high temperature 2 [11] and to the different degrees of population adaptation to heat [46]. In addition, local conditions, marked by income level [27], the population pyramid [34, 68] and the percentage of women [58], could account for the differing health effects of pollution.

The relationship between air pollution and emergency hospital admissions due to cardiovascular causes has been targeted by many studies, mostly at the level of single cities [9, 34, 38, 65], yet there are few studies that give a country-wide picture of how pollution affects this health indicator. Furthermore, when studies have examined the way in which temperature modifies the effects of NO_2_, PM_2.5_ and PM_10_ exposure on cardiorespiratory diseases, an increase in the impact of these pollutants during low-temperature episodes has been described [52]. In the case of O_3_ specifically, its atmospheric concentration tends to increase during high-temperature events since its formation is conditional upon the presence of sunlight [66]. It would therefore be plausible to expect its impact to be more pronounced during heat-wave episodes.

In addition to environmental variables, economic and demographic factors significantly condition the vulnerability of a given population to the adverse effects of climate change. There is a growing consensus in the literature to the effect that climate change does not affect all social groups equally, but instead actually magnifies pre-existing health inequalities, including access to health resources [54, 63]. People with lower income levels suffer from greater exposure to unhealthy environments, worse housing conditions and reduced capacity to adopt adaptive measures [49]. In parallel with this, the most vulnerable groups, like the elderly, face a higher risk of morbidity and mortality associated with air pollution and extreme temperatures, due to factors such as frailty and the presence of chronic diseases [3, 28]. In this context, these economic and demographic dimensions must be borne in mind when analyzing the impact of air pollutants and thermal extremes on hospital admissions, so as to be able to design more equitable and effective public health strategies.

The aim of this study was thus to analyse the short-term impact of the main air pollutants on emergency hospital admissions due to circulatory causes; and more specifically, to analyse their behaviour during extreme temperature episodes in Spain, determining specific dose–response functions for each place, cause of admission and air pollutant analysed. The goal was twofold: firstly, to give an overall picture of their relevance in terms of the total number of emergency admissions, with the aim of notably improving public health heat- and cold-wave prevention plans implemented in Spain in recent years; and secondly, to use the results obtained to contribute to the type of air-pollution control policies that have been reported to yield highly beneficial health effects in many countries.

## Methods

### Study Design

We performed a retrospective ecological time series analysis, evaluating the short-term effects that chemical air pollution and extreme temperatures in heat and cold waves have on emergency hospital admissions due to cardiovascular causes. The designated study period was 1 January 2013 to 31 December 2018. Spain is divided into 17 Autonomous Regions, which are in turn subdivided into a total of 52 provinces. These provinces were the basic study unit. The geographical distribution of these provinces is shown in Figure S1 of the supplementary material.

### Description of the Variables

#### Dependent Variables

The main dependent variable was the number of unscheduled daily admissions registered at hospitals across Spain. These data were sourced from the Hospital Morbidity Survey (*Encuesta de Morbilidad Hospitalaria*/*EMH*), whose sample included 95.4% of hospitals and 99.2% of hospital admissions in the country. The data were supplied by the National Statistics Institute (*Instituto Nacional de Estadística/INE*) under a confidential microdata assignment agreement. These daily emergency admission data at each hospital were grouped at a provincial level.

The causes of admission included in the study were all cardiovascular diseases (CVD) (ICD-9: 390–459; ICD-10: I00-I99), broken down into the following 3 specific causes: acute cerebrovascular disease (ACVA) (ICD-9: 431–434; ICD-10: I61-I64); acute myocardial infarction (AMI) (ICD-9: 410; ICD-10: I21-I22) and ischaemic heart disease (IHD) (ICD-9: 414.8, 414.9, 429.9; ICD-10: I51.9, I25.9).

#### Independent Variables

The independent variables consisted of chemical air pollution, meteorological and demographic and socio-economic variables.

The air pollution variables used were mean daily NO_2_, PM_10_, PM_2.5_ and O_3_ concentrations in g/m^3^. These variables were obtained by taking the values of the daily readings recorded and supplied by the Air Quality Surveillance Network of the Ministry for Ecological Transition & Demographic Challenge (*Ministerio para la Transición Ecológica y Reto Demográfico/MITECO*) and averaging them for each province. The Network includes 633 surveillance stations: of these, 286 monitor background levels, 130 monitor road traffic levels and 220 monitor industrial levels. In terms of location, 138 stations are rural, 214 are suburban and 284 are urban [43], as can be seen from Figure S1 of the supplementary material.

The quality of the records of these variables was tested by identifying missing values. All records having more than 10% of missing values were discarded for study purposes, in order to prevent any possible bias of representativeness. In all other cases, missing values were imputed by means of linear interpolation. The above quality control allowed us to include data on NO_2_ in 49 (94.2%), O_3_ in 48 (92.3%), PM_10_ in 44 (84.6%) and PM_2.5_ in 22 (42.3%) of the 52 provinces studied.

The meteorological variables used were the maximum daily temperature (T_max_) as the base definition of heat waves and the minimum daily temperature (T_min_) in the case of cold waves, since the respective threshold temperatures used by the Spanish Ministry of Health for its high- [39] and low-temperature prevention plans [40] are based on these. The data on these variables were measured and recorded at meteorological observatories representative of each province, and supplied by the State Meteorology Agency (*Agencia Estatal de Meteorología/AEMET*). In addition to these fundamental variables, we also used other meteorological control variables which previous studies had linked to health effects. Hence, we controlled for mean daily relative humidity [35], daily hours of sunlight [21], daily pressure trend [22] and mean daily wind speed [15]. These data were obtained from the same meteorological observatories as those used for temperature.

#### Economic and Demographic Variables

There are studies which report that income level [27], sex [58] and/or the population over the age of 64 years [34, 68] can influence the impact that air pollution has on health at any given place. In view of the fact that a small number of cases can cause a bias in the results of a study by becoming associated with higher attributable risks in the modelling results, these variables were taken into account to detect their possible influence on the percentage of cases attributable to pollution at a provincial level. The following independent variables were thus considered:

- *Population*: absolute number of census-registered inhabitants in the province
- *Income*: mean per capita income in the province
- *Population over 64*: population percentage aged 65 years and over in the province. The Spanish Ministry of Health’s High Temperature Prevention Plan [41] and Low Temperature Prevention Plan [42] specifically identify being over 65 as a personal risk factor
- *Women*: percentage of women in the province

These data were downloaded from the *INE*’s open data repository for 2015.

### Transformation of Variables

The first analysis performed was to determine the functional relationship between the independent variables and daily CVD-related admissions. These functional relationships were linear in all cases, with the exception of maximum daily temperature in heat waves, minimum daily temperature in cold waves, and in certain provinces, ozone concentrations, which display a quadratic or “U-shaped” relationship with mortality [17] and emergency hospital admissions [33, 56]. In order to pass from quadratic to linear relationships, these functions were parameterised and new variables created as follows:

$$\begin{array}{cc}O_{3high}\:=\:0&if\;O_3\:<\:O_{3threshold}\end{array}$$

$$\begin{array}{cc}O_{3high}\:=\:O_3-\;O{3threshold}_{3threshold}&if\;O_3\:>\:O_{3threshold}\end{array}$$

O_3threshold_ values were determined for each province in any case where the relationship was quadratic, and correspond to the vertex of the parabola. Table 1 shows the O_3threshold_ values that were obtained at a provincial level for cases in which the relationship was not linear.

**Table 1:** Thresholds applied for the Theat, Tcold and O_3high_ definitions: provincial level 2013–2018. Shown in brackets is the percentile to which the threshold concentration corresponds in the series of daily ozone concentrations

Autonomous region	Province	T_heat _threshold (°C)	T_cold _threshold (°C)	O_3high _threshold (μg/m^3^)
Andalusia	Almeria	35	6.2	90.6 (p88)
	Cadiz	38.5	2	82.8 (p80)
	Cordoba	41.5	1.5	Without threshold
	Granada	36.5	−0.7	Without threshold
	Huelva	38	5	81.3 (p79)
	Jaén	38.9	1.3	Without threshold
	Malaga	37.2	3.7	Without threshold
	Seville	40.5	4.4	82.5 (p92)
Aragon	Huesca	34.5	−0.8	67.3 (p55)
	Teruel	36.7	−6.7	Without threshold
	Zaragoza	38	0.4	Without threshold
Asturias	Asturias	26	−0.5	Without threshold
Balearic Islands	Balearics	33.3	5.9	Without threshold
Basque Country	Álava	33	−4.1	77.8 (p59)
	Bizkaia	33	0.2	70.2 (p89)
	Gipuzkoa	27.5	1.3	64.6 (p77)
Canary Islands	Las Palmas	33	13.2	Without threshold
	S.C. de Tenerife	34	13.8	Without threshold
Cantabria	Cantabria	26.5	0.3	47.8 (p34)
Castile-La Mancha	Albacete	37.6	−1.9	no data
	Ciudad Real	38	−0.1	Without threshold
	Cuenca	36	−2.5	90.0 (p91)
	Guadalajara	37	−2.8	no data
	Toledo	38	−0.8	93.8 (p94)
Castile & Leon	Avila	33	−4.2	Without threshold
	Burgos	33.5	−3.2	Without threshold
	Leon	33	−5.8	Without threshold
	Palencia	33	−5.7	69.4 (p60)
	Salamanca	35	−3.2	Without threshold
	Segovia	33.5	−1.9	Without threshold
	Soria	33.9	−5.7	79.0 (p86)
	Valladolid	36	−1.7	83.9 (p95)
	Zamora	37	−1.2	80.2 (p78)
Catalonia	Barcelona	31	3.8	Without threshold
	Girona	33.5	−0.2	87*(p86)
	Lleida	37.9	−2.9	93.0*(p95)
	Tarragona	35.5	0.2	Without threshold
Ceuta	Ceuta	33	8.7	no data
Extremadura	Badajoz	41	1.2	85.6 (p92)
	Cáceres	37	0.6	80.6* (p75)
Galicia	A Coruña (Corunna)	27.5	2	Without threshold
	Lugo	32.4	−5.6	Without threshold
	Ourense	37.4	1.5	Without threshold
	Pontevedra	28.5	4.8	Without threshold
La Rioja	La Rioja	34.5	−2.1	76.5* (p93)
Madrid	Madrid	34	1.9	93.6* (p96)
Melilla	Melilla	33.4	8	No data
Murcia	Murcia	38.8	3.3	75.4* (p65)
Navarre	Navarre	34	−3.7	Without threshold
Valencia	Alicante	32	4.4	Without threshold
	Castellón	32.5	4.5	Without threshold
	Valencia	34.5	2.9	Without threshold

In the case of the maximum daily temperature in heat waves (T_heat_) and minimum daily temperature in cold waves (T_cold_), the following variables were created:

$$\begin{array}{cc}T_{heat}\:=\:0&if\;T_{max}\:<\:T_{thresholdtmax}\end{array}$$

$$\begin{array}{cc}T_{heat}\:=\:T_{max}-T_{thresholdtmax}&if\;T_{max}\:<\:T_{thresholdtmax}\end{array}$$

$$\begin{array}{cc}T_{cold}\:=\:0&if\;T_{min}\:>\:T_{thresholdtmin}\end{array}$$

$$\begin{array}{cc}T_{cold}\:=\:T_{thresholdtmin}-T_{min}&if\;T_{min}\:<\:T_{thresholdtmin}\end{array}$$

In these equations, T_thresholdtmax_ refers to the heat wave definition threshold temperature at a provincial level used by the Spanish Ministry of Health for activation of the heat health-effect prevention plan [39], and T_thresholdtmin_ is the cold wave definition threshold temperature at a provincial level [40]. These values are shown in Table 1.

In addition, given that the effect of the variables considered may be lagged in time, we also considered the lags in each case.

### Other Control Variables

To control for possible autoregressive effects, we included 1st-order autoregressive variables of each dependent variable in the models. To control for seasonalities, we also included periodic variables generated on the basis of sine and cosine functions of the annual (365 days), six-monthly (180 days), four-monthly (120 days), quarterly (90 days), two-monthly (60 days) and monthly (30 days) periods. Similarly, as hospital admissions follow weekly seasonalities, the days of the week and Public Holidays were included as dummy variables.

### Determination of Relative Risks and Attributable Risks in the Dependent and Independent Variables

The effect associated with each risk factor was estimated using Poisson-family generalised linear models (GLMs), with the variables described above being used as dependent and independent variables. A model was fitted for each cause of admission and province studied. The strategy adopted for the design of the model was to include all the independent variables and their corresponding lags. Since there may be collinearity among the different independent variables, we used backward stepwise elimination, firstly eliminating variables of less statistical significance and so on successively. This process was repeated until all the variables in the model reached statistical significance (*p*-value < 0.05).

The coefficient which accompanies each of the variables that was shown to be significant in the GLM model, represents the relative risk (RR) factor associated with this variable via the equation RR = e^β^. Based on the RRs so obtained, we then estimated the attributable risks (ARs) in percentage terms, using Coste & Spira’s equation [12]:

$$\mathrm{AR}(\mathrm{\%})=\left(\frac{\mathrm{RR}-1}{\mathrm{RR}}\right)\times 100$$

Temperature-related risks were expressed for every 1 °C rise in the case of T_heat_ or every 1 °C drop in the case of T_cold_. In the case of pollutants, the risk was expressed for every increase of 10 g/m^3^.

### Calculation of Number of Daily Emergency Hospital Admissions Attributable to Air Pollution and Extreme Temperatures

Since the AR represents by how much CVD-related hospital admissions rise for every increase of 10 g/m^3^ in the mean daily concentration of a given pollutant, a simple proportion can be used to calculate the % increase corresponding to any particular pollutant concentration. This value would indicate the admissions attributable to that same pollutant if there were 100 admissions per day. Based on the real daily value of admissions due to CVD, one could then determine what proportion was specifically attributable to that pollutant.

In the case of T_heat_ and T_cold_ the procedure is the same, though taking into account the fact that in this case, the ARs are for increases of 1 °C.

### Influence of Economic and Demographic Variables

Lastly, we also examined whether the percentages of emergency CVD-related admissions in the respective provinces were related to income level and demographic composition. To this end, we fitted a random effects mixed model, using the percentages of emergency cardiovascular-cause admissions attributable to chemical air pollutants calculated in the above subsections as the dependent variable, and the variables described in the “Economic and Demographic Variables” section. as the independent variables. In the model, random effects were assigned to the provinces and to the chemical air pollutants responsible for the contribution of attributable admissions. We first used univariate models to analyse the effect of the socio-economic variables one by one, and then generated a model with all the variables co-adjusted.

All data-processing and statistical analyses were performed using the free R software suite version 4.4.2.

## Results

The descriptive statistics of the dependent variables and independent variables used in this study are shown in Tables S1 and S2 of the supplementary material respectively.

As stated in “Methods” section above, the functional relationships between the dependent (CVD) and independent variables proved to be linear in the case of NO_2_, PM_10_ and PM_2.5_ for all provinces. In the case of ozone, this functional relationship was quadratic in some provinces and linear in others. This vertex of the quadratic function can be used to define the mean daily concentrations of ozone above which there may be an impact on CVD-related hospital admissions (O_3high_). Shown in Table 1 are the values of the heat and cold wave threshold definition temperatures, along with the threshold values for the definition of the variable O_3high_.

The RRs and ARs at a provincial level, yielded by the modelling process for the independent variable and all the dependent variables analysed which proved to be statistically significant, are shown in Table S3 of the supplementary material.

**Fig. 1:**
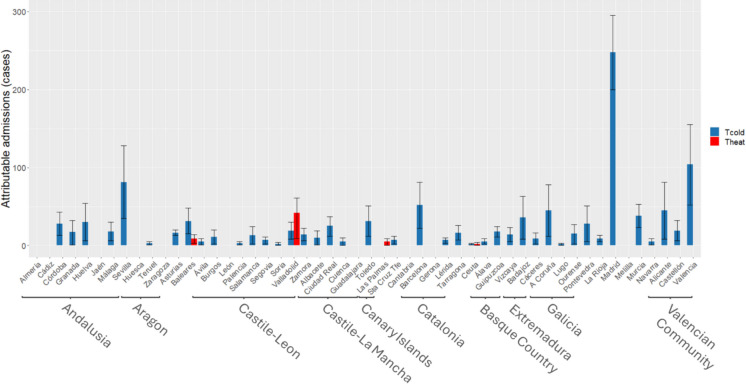
Annual number of hospital admissions attributable to heat and cold waves due to all cardiovascular causes (CVD) analysed for the respective Spanish provinces. The provinces are grouped by autonomous regions

**Fig. 2:**
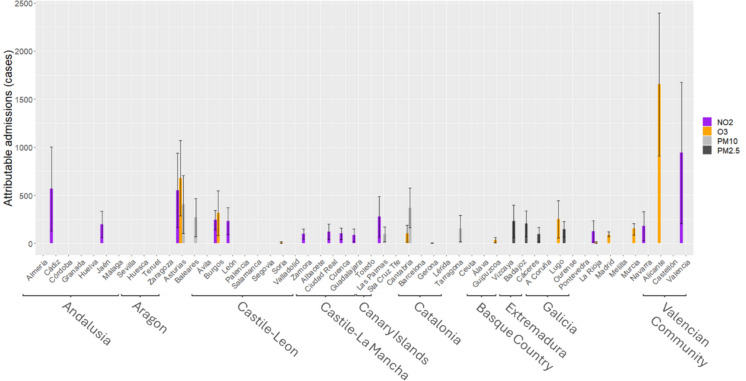
Annual number of hospital admissions attributable to the different air pollutants analysed. The provinces are grouped by autonomous regions

Based on these ARs, attributable cases were calculated at a provincial level for emergency hospital admissions due to CVD. Figure 1 shows the attributable cases for heat and cold waves at a provincial level. It should be noted here that the impact of cold waves was greater than that of heat waves in all provinces except Valladolid and that there were very few provinces in which the effect of heat waves was detected. Figure 2 shows the attributable cases due to CVD-related admissions for the different air pollutants analysed. Noteworthy here is the preponderant effect seen in the number of attributable cases and number of provinces which displayed an association with NO_2_ and O_3_ as opposed to PM. Note that both figures are at different scales in Y axis related to atributtable admissions and for the X axis province are sorted by autonomous community.

To obtain an overall picture of the number of attributable admissions due to CVD at the level of the various Autonomous Regions, these cases were grouped for the different variables analysed. The results are shown in Table 2, from which it will be observed that for Spain as a whole, the effect of NO_2_ and O_3_ was three times higher than that of PM. It is likewise noteworthy that the impact of heat was practically non-existent and that the effect of cold waves on emergency hospital admissions due to CVD was almost one order of magnitude lower than that attributable to air pollution.

**Table 2:** Annual number of hospital admissions due to cardiovascular causes (CVD), attributable to the respective variables analysed in Spain, by autonomous region

Autonomous region	NO_2_	O_3_	PM	T_cold_	T_heat_	Total air pollution	Total extreme temperature	Total
Andalusia	767 (189—1336)			174 (61–287)		767 (189–1336)	174 (61–287)	941 (250–1623)
Aragon				3 (1–5)			3 (1–5)	3 (1–5)
Asturias	552 (166–937)	679 (288–1069)	406 (106–706)	16 (13–20)		1637 (560–2712)	16 (13–20)	1653 (573–2732)
Balearic Isles			270 (72–465)	31 (15–48)	9 (3–14)	270 (72–465)	40 (18–62)	310 (90–527)
Castile & Leon	577 (279–867)	326 (86–565)		74 (22–125)	42 (9–61)	903 (365–1432)	116 (31–186)	1019 (396–1618)
Castile-La Mancha	311 (99–515)			71 (25–117)		311 (99–515)	71 (25–117)	382 (124–632)
Canary Islands	278 (64–487)		96 (20–173)	7 (2–12)	5 (0–9)	374 (84–660)	12 (2–21)	386 (86–681)
Cantabria		102 (14–191)	369 (163–575)			471 (177–766)		471 (177–766)
Catalonia		3 (0–4)	156 (18–292)	75 (32–117)		159 (18–296)	75 (32–117)	234 (50–413)
Ceuta				2 (1–3)	2 (0–4)		4 (1–7)	4 (1–7)
Euskadi (Basque Country)		34 (5–63)	231 (62–397)	37 (18–56)		265 (67–460)	37 (18–56)	302 (85–516)
Extremadura			303 (92–509)	45 (10–79)		303 (92–509)	45 (10–79)	348 (102–588)
Galicia		251 (56–444)	147 (66–226)	90 (19–159)		398 (122–670)	90 (19–159)	488 (141–829)
La Rioja	124 (13–236)	10 (1–19)		9 (5–13)		134 (14—255)	9 (5–13)	143 (19–268)
Madrid		92 (68–119)		248 (200–295)		92 (68–119)	248 (200–295)	340 (268–414)
Murcia		154 (85–206)		38 (23–53)		154 (85–206)	38 (23–53)	192 (108–259)
Barra	180 (26–331)			5 (1–9)		180 (26–331)	5 (1–9)	185 (27–340)
Valencia	945 (208–1674)	1653 (907–2394)		168 (66–268)		2598 (1115–4068)	168 (66–268)	2766 (1181–4336)
Total Spain	3734 (1044, 6383)	3304 (1510, 5074)	1978 (599, 3343)	1093 (514; 1666)	58 (5, 88)	9016 (3153–14,800)	1151 (526–1754)	10,167 (3679, 16,554)

**Fig. 3:**
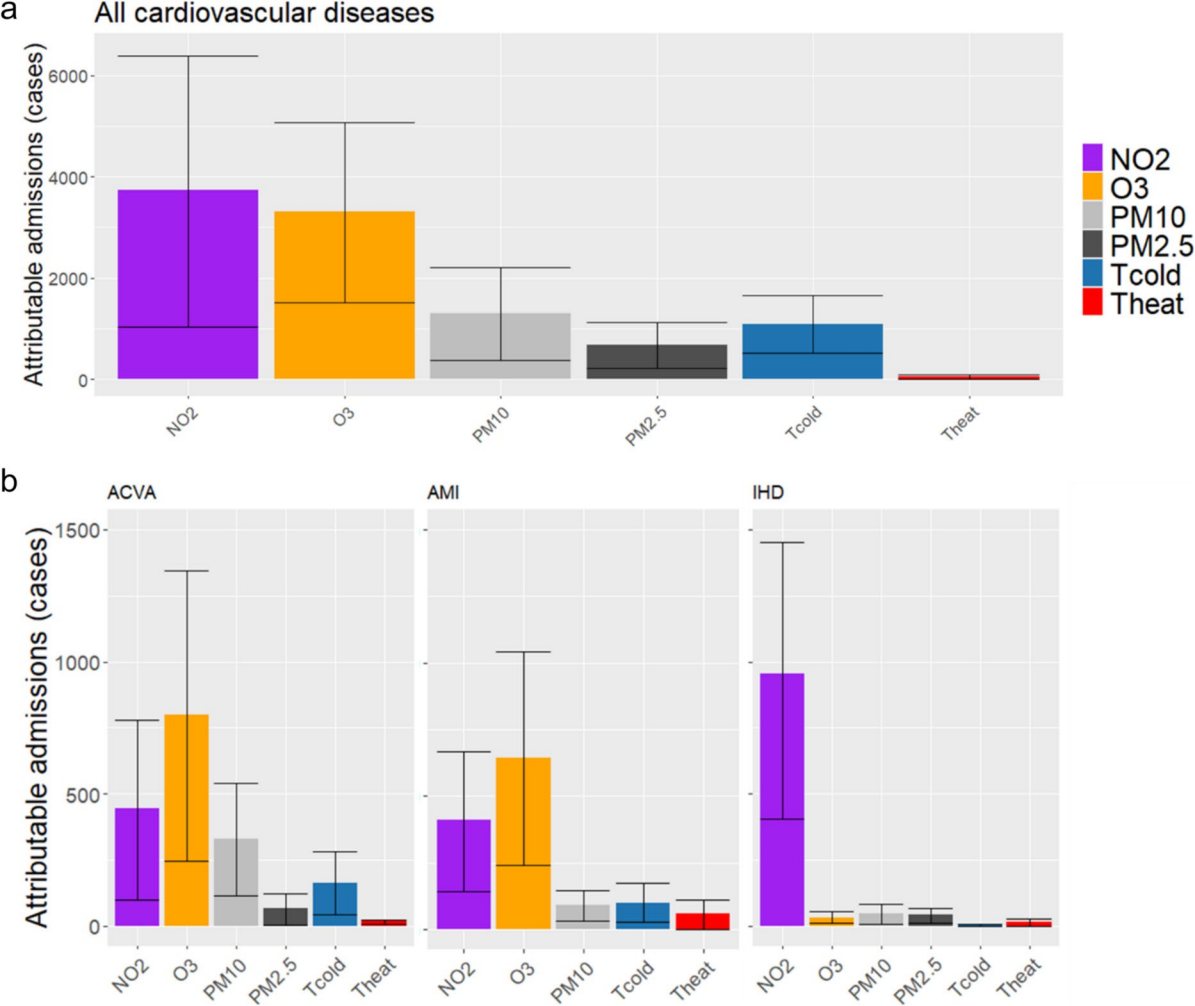
Admissions attributable to NO_2_, O_3_, PM_10_ and PM_2.5_, Tcold, Theat. Panel (**a**) shows results for all cardiovascular disease and panel (**b**) shows results for acute cardiovascular accident (ACVA), acute myocardial infarction (AMI) and ischaemic heart disease (IHD). There were no statistically significant effects of PM_2.5_ on AMI

Figure 3 shows annual attributable cases broken down by the different air pollutants for Spain overall, for all CVD (panel a), and for the different causes of admission analysed (panel b). From this, it will be observed that the effects of NO_2_ and O_3_ were mutually similar and very much greater than that of PM. Only in the case of IHD was the effect of NO_2_ very much greater than that of the remaining pollutants. Figure 3 also shows the effects of the heat and cold waves, both for all CVD and for the specific cardiovascular causes analysed. As can be seen from this figure, the effect of cold waves was greater than that of heat waves on all causes analysed, except in the case of ischaemic disease, in which both were similar (without any statistically significant difference).

Based on the percentage of attributable CVD-related admissions due to chemical air pollution in each province, taking into account the demographic and economic variables in each province (number of inhabitants, population percentage over 64 years of age, percentage of women and per capita income level), and using the random effects mixed-model method, the simple model showed that the population over the age of 64 constituted a risk factor, with a 15% (95%CI: 9–20) increase in the attributable risk of admission due to CVD for each percentage point of the over-64 population. In the models coadjusted for the 4 variables considered, the income level was shown to act as a protective factor against CVD-related admissions, such that an increase of €10,000 in per capita income was related with a 5% decrease (95%CI: 10–0) in the risk of attributable admissions due to CVD.

## Discussion

The data in Table S1 (supplementary material) show that there are 133,000 emergency hospital admissions due to CVD in Spain annually. According to the results obtained in Table 2, 10,200 of these admissions (7.7%) can be assumed to be related with the variables analysed; and of these, 6.9% would be attributable to air pollution and 0.8% to heat and cold waves. This figure of 6.9% is slightly lower than that reported by another study undertaken in Spain targeting short-term admissions due to respiratory diseases [33], which established that admissions attributable to air pollution alone account for 7.8% of total admissions occurring in Spain due to the above cause. At the EU level, there are very few studies analysing the effect of air pollution on cardiovascular morbidity. Perhaps the most comprehensive is the Eionet Report [57], which calculates the years lived with disability (YLD) attributable to stroke burden disease (ICD- 10: I60-I69) for people over 25 years of age in all EU-27 countries. According to this study, the YLD/10^5^ inhabitants for Spain attributable to PM_2.5_ would be 17.1 CI 95%: (1.9–32.6), while the value for the EU 27 would be 35.7 CI95%: (4.0–65.9). In other words, Spain would be below the EU 27 average. The same is true for NO_2_, with a YLD/10^5^ inhabitants value of 8.6 CI 95%: (4.5 12.4), while the value for the EU 27 would be 12.0 CI 95: (6.2 17.3). However, it should be noted that the Eionet Report only analyses morbidity associated with stroke and not that due to all cardiovascular causes, as is the case in our research. It also does not consider the effect of PM_10_ or O_3_.

Furthermore, total hospital admissions attributable to air pollution in Spain are estimated at a figure of 62,000 admissions/year [56], so that the 9000 CVD-related admissions obtained in this study would represent 14.5% of all admissions attributable to pollution in this country. This figure is considerably lower than that of 53.3% relating to respiratory cause admissions due to air pollution in Spain [33], and coincides with the figure found in other studies which report that the greatest disease burden attributable to air pollution is due to respiratory causes [30], specifically in short-term hospital admissions in Mediterranean Europe [59].

### Effect of Heat and Cold Waves

The most noteworthy result as regards the effect of extreme temperatures on short-term hospital admissions due to CVD, is that high temperatures in heat waves have practically no impact on emergency hospital admissions due to cardiovascular causes (Fig. 1). This same result was obtained by a further study conducted in Spain on the effect of heat on different causes of admission, which concluded that this effect is very slight in comparison with other causes of admission, such as renal or endocrine causes [1]. Similar results were obtained by another study undertaken in the USA on the impact of heat waves on hospital admissions, which reported that there was an association between respiratory and renal causes, but not with CVD [26].

This lack of association between heat wave temperatures and cardiovascular-cause admissions seems to be at variance with the biological mechanisms associated with high temperatures. High temperatures can increase platelet and red cell count, blood viscosity and plasma cholesterol level during heat stress, as well as mortality from coronary and cerebral thrombosis [29, 50], and an association should therefore be detected with admissions due to circulatory diseases, which is not at all the case in our study. The only possible explanation would appear to be that the above pathologies involve rapidly fatal health outcomes with a short-time interval between exposure to high temperature and death then not be counted within the hospital admissions figures [31, 37].

Although the effect of temperature extremes on hospital admissions is small compared to that of air pollution, some studies analysing the future evolution of morbidity related to temperature extremes suggest that this effect will increase. Mortality will decrease due to fewer cold extremes. These results underscore the need to quantify temperature-morbidity responses to fully understand and anticipate the health impacts of climate change and suggest that local declines in mortality due to warming can mask economically meaningful increases in temperature-driven morbidity and healthcare utilisation [23].

Figure 3 shows that the effect of cold waves on attributable admissions for both CVD and the specific causes analysed (except for IHD) is greater than that of heat waves. Other studies carried out in Spain on emergency hospital admissions due to all causes [56] and to respiratory causes [33] report a greater effect for cold waves than for heat waves. In both cases, the proportion of admissions attributable to cold waves is 3 times higher than that attributable to heat waves. In the case of admissions due to CVD, however, this proportion is 19 times higher. This finding, which is not supported in the case of mortality on which the two impacts are similar [16], bolsters the above hypothesis of the immediacy of cardiovascular processes, in that there are studies which show that cold also brings about changes in blood clotting, linking these to an increase in myocardial infarction and thrombosis [13], as well as a rise in blood pressure, cholesterol, fibrinogens and number of erythrocytes [62], though with effects that are not caused quite as promptly [15].

#### Effect of Air Pollution

The scientific evidence shows that even low levels of exposure to air pollution are associated with negative health impacts [6, 19, 60], such as increased morbidity and mortality due to circulatory causes [24]. Several mechanisms related to air pollution are involved in cardiovascular disease, mainly the risk of atherothrombosis [5, 53]. Some studies suggest that exposure to air pollution may increase blood pressure, exacerbate myocardial ischaemia and trigger myocardial infarction. Many of these effects may be mediated through direct or indirect effects on the systemic vasculature, driven by endothelial dysfunction [47]. Although many of the studies into the effect of pollution focus on the effect of PM [38, 47, 53], other studies also link NO_2_ concentrations to a higher number of admissions due to cardiovascular diseases [9], establishing increases in risk ranging from 2.8% for myocardial infarction to 4.9% for haemorrhagic stroke. The results of this study reinforce the evidence of the short-term effects of NO_2_ on hospital admissions due to cardiovascular disease. The different lag between exposure and a health-related event for haemorrhagic stroke as compared to ischaemic stroke suggests different mechanisms of action. Other studies link ozone to different causes of admission due to cardiovascular diseases, indicating the existence of a threshold value. Levels of 100 μg/m^3^ or higher were associated with substantial increases in hospital admissions for cardiovascular disease, ranging from 3.38% for stroke to 6.52% for acute myocardial infarction. Nevertheless, lower concentrations of 70 to 99 μg/m^3^ (vs. below 70 μg/m^3^) were also linked to increases in hospital admissions, ranging from 2.26% for heart failure to 3.21% for coronary heart disease [45]. These results would be in line with the determination in some provinces of a threshold level for ozone concentrations, above which their effect on health clearly increases.

Different studies into the joint effect of several pollutants on short-term CVD admissions show that the highest risks are observed for NO_2_, followed by PM, and lastly, ozone, though the differences are not statistically significant [34]. Similar results are reported in another study also conducted in a city in China [65].

Nitric oxide is a ubiquitous, naturally occurring molecule found in a variety of cell types and organ systems. In the cardiovascular system, nitric oxide is an important determinant of basal vascular tone, prevents platelet activation, limits leukocyte adhesion to the endothelium and regulates myocardial contractility. Nitric oxide may also play a role in the pathogenesis of common cardiovascular disorders, including hypotension accompanying shock states, essential hypertension, and aterosclerosis [36]. Each of the major risk factors predisposing to vascular disease are associated with endothelial cell dysfunction, suggesting a direct etiologic link between the effects of the risk factors on the endothelium and their propensity to accelerate vascular disease. Restoration or replacement of endothelium-derived factors such as nitric oxide which impede the progression of vascular disease, or preventing the action of mediators such as vasoconstrictor eicosanoids, angiotensin II or endothelin, which accelerate the progression of vascular disease [8].

The results obtained, which are depicted in Fig. 2, indicate that for Spain as whole the pollutant that displays the relationship with the highest number of emergency attributable admissions due to CVD and the specific causes considered is NO2, followed by ozone, with PM showing the weakest association, though in the majority of cases these differences are not statistically significant. This lower effect of PM in respect of emergency admissions in Spain has been observed in analyses performed for all causes [56] and for respiratory causes [33], as well as in studies at a provincial level [56], and might be related to the marked reduction in PM_10_ and PM_2.5_ observed across the study period in Spain, especially in urban areas, something that does not occur with ozone concentrations which have in fact experienced a rise in urban areas [44].

On the other hand, the effect that air pollution has on attributable hospital admissions is one order of magnitude higher than that of extreme temperatures (9000 vs. 1150 cases/year). This may be due to the fact that the number of days per year with exposure to a heat or cold wave is lower than the number of days with exposure to air pollution alone, as can be seen in Table S2 of the supplementary material. This does not imply that on days on which there is a heat or cold wave, the effect of temperature on hospital admissions is not important, but rather that over the year as a whole the effect of pollution is greater than that of temperature. That said, studies on the morbidity and mortality impact of temperature and air pollution in heat waves [55] indicate that, while the effect of temperature is important, the effect of air pollution is at least similar. The same occurs in the case of cold waves [14]. Hence, prevention plans seeking to minimise the impacts of extreme temperatures on health should, at minimum, integrate both effects [32].

#### Demographic and Economic Modelling Variables

The results of the random effects mixed model fitted to observe the possible effect of demographic and socio-economic variables on the percentage of attributable admissions in each province show that in those provinces where the population percentage aged 65 years and over is high, the effect of air pollution on CVD-related admissions tends to be greater. This is only logical since air pollution exacerbates previous diseases [68], and it is in this age group that the number of existing diseases is highest. Even so, other studies indicate that the effect of NO_2_ on CVD-related admissions is greatest in the under-65 age group [34, 65]. While some studies have found a greater effect of air pollution, and NO_2_ in particular, on CVD-related admissions among women in some cities [34, 65], the results of our study at a national level do not allow us to show that the percentage of women might be a risk factor in terms of hospital admissions due to CVD. This finding is in line with those reported by other studies, which indicate that there is no conclusively clear distinction by sex between the effects of pollution since sex-related differences in the effect of ambient air pollution varied across health outcomes, causes, seasons and times [58].

The income level in the mixed model coadjusted for all the variables, showed itself to be a protective factor against hospital admissions due to CVD, inasmuch as provinces with the highest income levels register a lower percentage of admissions attributable to air pollution. A study led by Brekel et al. [7] established that places with lower income levels in The Netherlands are subjected to higher levels of exposure to pollution and, thus, to greater expected impacts on the health of exposed individuals. Our results seem to confirm these greater expected impacts on health in more deprived Spanish provinces, although the design of the mixed model does not allow us to confirm if that result is due to higher exposure, higher vulnerability or both.

Whereas North American studies have shown that areas where low socio-economic status communities dwell experience higher concentrations of criteria air pollutants, European research has had mixed results. In contrast, research from Asia, Africa and other parts of the world has shown a general trend similar to that of North America [27].

### Study Limitations

In addition to the limitations inherent in ecological studies, which do not allow for causality to be inferred or assumptions made at an individual level, this study has limitations related with the assignment of exposure to the different environmental variables. Previous studies have established that pollution readings taken at official monitoring stations may underestimate the real immission levels to which the population is subjected. It should be borne in mind that air-quality monitoring station networks, at least in Europe, are made up of a higher number of rural and urban background stations than urban and suburban stations focusing on road traffic or major industrial foci [20]. Hence, the mean levels recorded by these networks would largely represent background levels of air pollutants for a given area, which are lower than those to which a person might be exposed in the vicinity of the principal sources of these pollutants in urban environments.

Similarly, maximum daily temperatures were monitored at meteorological stations located at some distance from the real place of exposure. These misalignment problems are intrinsic to these types of ecological studies [4]. However, the study design enables the scope of this limitation to be reduced, i.e. on working with aggregate data, it can be assumed that the data used are representative of the average exposure experienced by the population.

There may also be biases associated with erroneous classification of the underlying cause of hospitalisation. Incorrect classification of the cause of admission is an inherent bias in studies that use diagnostic codes, in which there may be different comorbidities [64]. In the case of cardiovascular diseases, this may be due to several factors, including the complexity of heart diseases and variability in the interpretation of diagnostic codes. Due to the high number of cases included in this study and its random nature, it is not expected to affect the results obtained.

In this study, in which specific dose–response functions calculated at a provincial level were used, the association was only taken into account when it was statistically significant, i.e. by virtue of being attributable to a given pollutant that remained at the limit of significance, there may have been cases of admission in which this study deemed there was no effect. This bias would be minimising the number of attributable admissions obtained here. In addition, by using specific causes of admission, the low number of daily cases in some provinces could lead to the RRs and ARs obtained being increased, though the fact that the variable “population” did not prove to be significant in the random effects mixed models renders this potential bias of no significance.

The use of data corresponding to hospitalised patients may produce a Berkson bias. Berkson’s bias can occur when hospital controls are used in a case–control study. If controls are hospitalised due to an exposure that is also related to the health outcome under study, the effect measure may be weakened, i.e. biased towards the null hypothesis of no association. In this case, as it is an ecological study of the entire population, Berkson’s bias is practically null and could only influence those patients who have already been hospitalised previously for cardiovascular disease. This information is not available in our study as it is aggregate data. Furthermore, no specific validation was carried out to assess the representativeness of the spatial variability of air pollutants, so our study also suffered from a measurement error of the Berkson type, among other biases associated with ecological exposure, as is common in most time series studies on air pollution, which does not give rise to any bias or only a very small one, but reduces statistical power.

## Conclusions

Every year, air pollution in Spain is linked to a total of 9000 attributable emergency admissions due to cardiovascular causes, whereas those attributable to heat and cold waves scarcely reach 1200. These short-term admissions attributable to pollution account for 6.9% of all emergency admissions in Spain due to cardiovascular causes. The principal pollutants linked to these admissions are NO_2_ and O_3_. NO_2_ is a primary pollutant, fundamentally emitted in urban atmospheres by road traffic, while O_3_ is a secondary pollutant, whose main precursors are NOx. Hence, measures targeted at reducing road traffic, particularly in urban agglomerations, would result in a decrease in admissions due to this cause. Chief among such measures would be the implementation of Low Emission Zones in cases where the law makes provision for these, the fostering of sustainable public transport and the promotion of the use of alternative mobility options.

Following the recomendations of European Society of Cardiology [47], the measures which could be implemented in the current and future policy about air pollution in the different Spanish provinces to protect the cardiovascular health in more vulnerable population should be base on people with or at high risk of cardiovascular disease should be informed about these measures to limit exposure to pollutants; public transportation should be preferred over driving; avoid walking and cycling on busy streets, especially during rush hour; exercise in parks and gardens, but avoid main roads and limit time spent outdoors during periods of high pollution, especially for infants, the elderly and people with cardiorespiratory disorders.

## Supplementary Information

Below is the link to the electronic supplementary material.

Supplementary Material 1 (DOCX 238 KB)

## Declarations

### Disclaimer

The views expressed by the authors are not necessarily those of the institutions with which they are affiliated.

## References

[1] Achebak H, Rey G, Chen ZY, Lloyd SF, Quijal-Zamorano M, Méndez-Turrubiates RF, Ballester F. Heat exposure and cause-specific hospital admissions in Spain: a nationwide cross-sectional study. *Environ Health Perspect*. 2024;132(5): 057009. 38775486 10.1289/EHP13254 PMC11110655

[2] Al Ahad MA, Sullivan F, Demšar U, et al. The effect of air-pollution and weather exposure on mortality and hospital admission and implications for further research: a systematic scoping review. *PLoS One*. 2020;15: e0241415. 33119678 10.1371/journal.pone.0241415 PMC7595412

[3] Atwoli L, Baqui AH, Benfield T, Bosurgi R, Godlee F, Hancocks S, Horton R, Laybourn-Langton L, Monteiro CA, Norman I, Patrick K, Praities N, Olde Rikkert MGM, Rubin EJ, Sahni P, Smith R, Talley NJ, Turale S, Vázquez D. Call for emergency action to limit global temperature increases, restore biodiversity, and protect health. *Lancet*. 2021;398(10304):939–41. 10.1016/S0140-6736(21)01915-2. 34496267 10.1016/S0140-6736(21)01915-2 PMC8428481

[4] Barceló MA, Varga D, Tobias A, Diaz J, Linares C, Saez M. Long term effects of traffic noise on mortality in the city of Barcelona, 2004–2007. *Environ Res*. 2016;147:193–206. 26894815 10.1016/j.envres.2016.02.010

[5] Bhatnagar A. Environmental cardiology. Studying mechanistic links between pollution and heart disease. *Circ Res*. 2006;99:692–705. 17008598 10.1161/01.RES.0000243586.99701.cf

[6] Boogaard H, Crouse DL, Tanner E, Mantus E, van Erp AM, Vedal S, et al. Assessing adverse health effects of long-term exposure to low levels of ambient air pollution: the HEI experience and what’s next? *Environ Sci Technol*. 2024;58(29):12767–83. 38991107 10.1021/acs.est.3c09745 PMC11270999

[7] Brekel Van den, Lieke et al. Ethnic and socioeconomic inequalities in air pollution exposure: a cross-sectional analysis of nationwide individual-level data from the Netherlands. *Lancet Planet Health*. 2024;8:e18–e29. 10.1016/S2542-5196(23)00258-9 38199717

[8] Cohen RA. The role of nitric oxide and other endothelium-derived vasoactive substances in vascular disease. *Prog Cardiovasc Dis*. 1995;38(2):105–28. 7568902 10.1016/s0033-0620(05)80002-7

[9] Collart P, Dubourg D, Levêque A, Sierra NB, Coppieters Y. Short-term effects of nitrogen dioxide on hospital admissions for cardiovascular disease in Wallonia, Belgium. *Int J Cardiol*. 2018;255:231–6. 29288056 10.1016/j.ijcard.2017.12.058

[10] Comisión Europea. Directiva del Parlamento Europeo y del Consejo sobre la calidad del aire ambiente y a una atmósfera más limpia en Europa. *PE-CON*S 88/24. Bruselas. 2024. https://data.consilium.europa.eu/doc/document/PE-88-2024-INIT/es/pdf

[11] Conti A, Valente M, Paganini M, Farsoni M, Ragazzoni L, Barone-Adesi F. Knowledge gaps and research priorities on the health effects of heatwaves: a systematic review of reviews.* Int J Environ Res Public Health*. 2022;19(10): 5887. 35627424 10.3390/ijerph19105887 PMC9140727

[12] Coste J, Spira A. Proportion of cases attributable to public health: definition(s), estimation(s) and interpretation. *Rev Epidemiol Sante Publique*. 1991;39(4):399–411. 1754705

[13] Davidkovova H, Plavcova E, Kyncl J, Kysely J. Impacts of hot and cold spells differ for acute and chronic ischaemic heart diseases. *BMC Public Health*. 2014;14: 480. 24886566 10.1186/1471-2458-14-480 PMC4038364

[14] Díaz J, Alberdi JC, Pajares MS, López R, López C, Otero A. A model for forecasting emergency hospital admissions: effect of environmental variables. *J Environ Health*. 2001;64:9–15. 11605333

[15] Díaz J, García R, Prieto L, López C, Linares C. Mortality impact of extreme winter temperatures. *Int J Biometeorol*. 2005;49:179–83. 15290433 10.1007/s00484-004-0224-4

[16] Díaz J, Carmona R, Mirón IJ, Ortiz C, Linares C. Comparison of the effects of extreme temperatures on daily mortality in Madrid (Spain), by age group: the need for a cold wave prevention plan. *Environ Res*. 2015;2015(143):186–91. 10.1016/j.envres.2015.10.018 26496852

[17] Díaz J, Ortiz C, Falcón I, Linares C. Short-term effect of tropospheric ozone on daily mortality in Spain. *Atmos Environ*. 2018;2018(187):107–16.

[18] Dirección General de Medio Ambiente (Comisión Europea), Kantar. Attitudes of Europeans towards the environment: Report [Internet]. Oficina de Publicaciones de la Unión Europea; 2020. Available from: https://data.europa.eu/doi/10.2779/902489. Accessed 2 Sep 2024.

[19] Dominici F, Zanobetti A, Schwartz J, Braun D, Sabath B, Wu X. Assessing adverse health effects of long-term exposure to low levels of ambient air pollution: implementation of causal inference methods. *Res Rep Health Eff Inst*. 2022;2022:211. 36193708 PMC9530797

[20] EEA. European Environment Agency. Air quality in Europe — 2020. 2020. EEA Report 09/2020, 160 pp. 10.2800/786656.

[21] Egea A, Linares C, Díaz J, Gómez L, Calle A, Navas MA, Ruiz-Páez R, Asensio C, Padrón-Monedero A, López-Bueno JA. How heat waves, ozone and sunlight hours affect endocrine and metabolic diseases emergency admissions? A case study in the Region of Madrid (Spain). *Environ Res*. 2023;229: 116022. 10.1016/j.envres.2023.116022 37121348

[22] González S, Díaz J, Pajares MS, Alberdi JC, López C, Otero A. Relationship between atmospheric pressure and mortality in the Madrid Autonomous Region: a time series study. *Int J Biometeorol*. 2001;45:34–40. 11411413 10.1007/s004840000076

[23] Gould CF, Heft-Neal S, Heaney AK, Bendavid E, Callahan CW, Kiang MV, et al. Temperature extremes impact mortality and morbidity differently. *Sci Adv*. 2025;11:eadr3070. 10.1126/sciadv.adr3070 PMC12309665 40737397

[24] Gouveia N, Rodriguez-Hernandez JL, Kephart JL, Ortigoza A, Betancourt RM, Sangrador JLT, et al. Short-term associations between fine particulate air pollution and cardiovascular and respiratory mortality in 337 cities in Latin America. *Sci Total Environ*. 2024;920:171073. 10.1016/j.scitotenv.2024.171073 PMC10918459 38382618

[25] Grigorieva E, Lukyanets A. Combined effect of hot weather and outdoor air pollution on respiratory health: literature review. *Atmosphere*. 2021;12:790.

[26] Gronlund CJ, Zanobetti A, Schwartz JD, Wellenius GA, O´Neill MS. Heat, heat waves, and hospital admissions among the elderly in the United States, 1992–2006. *Environ Health Perspect*. 2014;122(11):1187–92. 24905551 10.1289/ehp.1206132 PMC4216145

[27] Hajat A, Hsia C, O’Neill MS. Socioeconomic disparities and air pollution exposure: a global review.* Curr Environ Health Rep*. 2025;2(4):440–50. 10.1007/s40572-015-0069-5 PMC4626327 26381684

[28] Intergovernmental Panel on Climate Change (IPCC). Climate Change 2021 – The Physical Science Basis: working Group I Contribution to the Sixth Assessment Report of the Intergovernmental Panel on Climate Change. Cambridge: Cambridge University Press; 2023.

[29] Keatinge WR, Coleshaw SR, Easton JC, et al. Increased platelet and red cell counts, blood viscosity, and plasma cholesterol level during heat stress, and mortality from coronary and cerebral thrombosis. *Am J Med*. 1986;81:795–800. 10.1016/0002-9343(86)90348-7 3776986

[30] Landrigan PJ, Fuller R, Acosta NJR, Adeyi O, Arnold R, Basu NN, Baldé AB, Bertollini R, Bose-O’Reilly S, Boufford JI, Breysse PN, Chiles T, Mahidol C, Coll-Seck AM, Cropper ML, Fobil J, Fuster V, Greenstone M, Haines A, Hanrahan D, Hunter D, Khare M, Krupnick A, Lanphear B, Lohani B, Martin K, Mathiasen KV, McTeer MA, Murray CJL, Ndahimananjara JD, Perera F, Potočnik J, Preker AS, Ramesh J, Rockström J, Salinas C, Samson LD, Sandilya K, Sly PD, Smith KR, Steiner A, Stewart RB, Suk WA, van Schayck OCP, Yadama GN, Yumkella K, Zhong M. The lancet commission on pollution and health. *Lancet*. 2018;391(10119):462–512. 10.1016/S0140-6736(17)32345-0 29056410

[31] Linares C, Díaz J. Impact of high temperatures on hospital admissions: comparative analysis with previous studies about mortality (Madrid). *Eur J Public Health*. 2007;18:318–22. 10.1093/eurpub/ckm108 18045814

[32] Linares C, Sanchez-Martinez G, Kendrovski V, Diaz J. A new integrative perspective on early warning systems for health in the context of climate change. *Environ Res*. 2020;187: 109623. 32416361 10.1016/j.envres.2020.109623

[33] Linares C, Díaz J, Navas MA, Ruiz-Páez R, Saez M, Barceló MA, López-Bueno JA. How air pollution and extreme temperatures affect emergency hospital admissions due to various respiratory causes in Spain, by age group: a national study.* Int J Hyg Environ Health*. 2025;266:114570. 10.1016/j.ijheh.2025.114570 40138959

[34] Liu Y, Guo M, Wang J, Gong Y, Huang C, et al. Effect of short-term exposure to air pollution on hospital admission for cardiovascular disease: a time-series study in Xiangyang, China. *Sci Total Environ*. 2024;918: 170735. 10.1016/j.scitotenv.2024.170735 38325454

[35] López-Bueno JA, Díaz J, Padrón-Monedero A, Navas Martín MA, Linares C. Short-term impact of extreme temperatures, relative humidity and air pollution on emergency hospital admissions due to kidney disease and kidney-related conditions in the Greater Madrid area (Spain). *Sci Total Environ*. 2023;903:166646. 10.1016/j.scitotenv.2023.166646. 10.1016/j.scitotenv.2023.166646 37652385

[36] Loscalzo J, Welch G. Nitric oxide and its role in the cardiovascular system. *Prog Cardiovasc Dis*. 1995;38(2):87–104. 7568906 10.1016/s0033-0620(05)80001-5

[37] Mastrangelo G, Hajat S, Fadda E, et al. Contrasting patterns of hospital admissions and mortality during heat waves: are deaths from circulatory disease a real excess or an artefact? *Med Hypotheses*. 2006;66:1025–8. 16413137 10.1016/j.mehy.2005.09.053

[38] Maté T, Guaita R, Pichiule M, Linares C, Díaz J. Short-term effect of fine particulate matter (PM2.5) on daily mortality due to diseases of the circulatory system in Madrid (Spain). *Sci Total Environ*. 2010;408:5750–7. 20825976 10.1016/j.scitotenv.2010.07.083

[39] Minsan. Ministerio de Sanidad. Plan Nacional de actuaciones preventivas por altas temperaturas 2023–2024. 2023a. https://www.sanidad.gob.es/areas/sanidadAmbiental/riesgosAmbientales/calorExtremo/publicaciones/informesAnteriores/docs/Balance_Plan_Calor_2023.pdf

[40] Minsan. Ministerio de Sanidad. Plan Nacional de actuaciones preventivas por bajas temperaturas 2023–2024. 2023b.https://www.sanidad.gob.es/areas/sanidadAmbiental/riesgosAmbientales/frioExtremo/publicaciones/docs/Plan_Frio_23-24.pdf

[41] Minsan. Ministerio de Sanidad. Plan Nacional de actuaciones preventivas por altas temperaturas 2025. 2025. https://www.sanidad.gob.es/areas/sanidadAmbiental/riesgosAmbientales/calorExtremo/publicaciones/docs/planNacionalExcesoTemperaturas_2025.pdf. Accessed June 2025.

[42] Minsan. 2024. https://www.sanidad.gob.es/areas/sanidadAmbiental/riesgosAmbientales/frioExtremo/publicaciones/docs/Plan_Frio_24-25.pdf. Accessed Apr 2025.

[43] MITECO. Redes de vigilancia de la calidad del aire. 2024. https://www.miteco.gob.es/es/calidad-y-evaluacion-ambiental/temas/atmosfera-y-calidad-del-aire/calidad-del-aire/evaluacion-datos/redes.html. Accessed Jan 2025.

[44] MITECO (2023). Tendencias de la calidad del aire en España 2001–2021. https://www.miteco.gob.es/content/dam/miteco/es/calidad-y-evaluacion-ambiental/temas/atmosfera-y-calidad-del-aire/analisisdetendenciasdelosprincipalescontaminantesatmosfericos_tcm30-561228.pdf. Accessed Jan 2025.

[45] Münzel T, Hahad O, Daiber A. The emergence of the air pollutant ozone as a significant cardiovascular killer? *Eur Heart J*. 2023. 10.1093/eurheartj/ehad046. 10.1093/eurheartj/ehad046 36893806

[46] Navas-Martín, M Á, Ovalle-Perandones MA, López-Bueno J A, Díaz J, Linares C, Sánchez-Martínez G. Population adaptation to heat as seen through the temperature-mortality relationship, in the context of the impact of global warming on health: a scoping review. *Sci Total Environ*. 2024;908:168441. 10.1016/j.scitotenv.2023.168441. 10.1016/j.scitotenv.2023.168441 37949135

[47] Newby DE, Mannucci P, Tell G, Baccarelli AA, Brook RD, Donaldson K, Forastiere F, Franchini M, Franco OH, Graham I, Hoek G, Hoffmann B, Hoylaerts MF, Künzli N, Mills N, Pekkanen J, Peters A, Piepoli MF, Rajagopalan S, Storey R, ESC Working Group on Thrombosis, European Association for Cardiovascular Prevention and Rehabilitation and ESC Heart Failure. Expert position paper on air pollution and cardiovascular disease. *Eur Heart J*. 2015;36:83–93. 25492627 10.1093/eurheartj/ehu458 PMC6279152

[48] Ni J, Zhao Y, Li B, et al. Investigation of the impact mechanisms and patterns of meteorological factors on air quality and atmospheric pollutant concentrations during extreme weather events in Zhengzhou city, Henan Province. *Atmos Pollut Re*s. 2023;14: 101932.

[49] Paavola J. Health impacts of climate change and health and social inequalities in the UK. Environmental Health: a Global Access Science Source. 2017;16(1):61–8. 10.1186/S12940-017-0328-Z. 10.1186/s12940-017-0328-z PMC5773866 29219089

[50] Pan WH, Li LA, Tsai MJ. Temperature extremes and mortality from coronary heart disease and cerebral infarction in elderly Chinese. *Lancet*. 1995;345:353–5. 7845116 10.1016/s0140-6736(95)90341-0

[51] Park K, Jin HG, Baik JJ. Do heat waves worsen air quality? A 21-year observational study in Seoul, South Korea. *Sci Total Environ*. 2023;884: 163798. 37127155 10.1016/j.scitotenv.2023.163798

[52] Parry M, Green D, Zhang Y, Hayen A. Does particulate matter modify the short-term association between heat waves and hospital admissions for cardiovascular diseases in greater Sydney, Australia? *Int J Environ Res Public Health*. 2019;3270(16):3270. 10.3390/IJERPH16183270. 10.3390/ijerph16183270 PMC6765779 31492044

[53] Rajagopalan S, Al-Kindi SG, Brook RD. Air pollution and cardiovascular disease. *J Am Coll Cardiol*. 2018;72:2054–70. 30336830 10.1016/j.jacc.2018.07.099

[54] Romanello M, McGushin A, di Napoli C, Drummond P, Hughes N, Jamart L, Kennard H, Lampard P, Solano Rodriguez B, Arnell N, Ayeb-Karlsson S, Belesova K, Cai W, Campbell-Lendrum D, Capstick S, Chambers J, Chu L, Ciampi L, Dalin C, Dasandi N, Dasgupta S, Davies M, Dominguez-Salas P, Dubrow R, Ebi KL, Eckelman M, Ekins P, Escobar LE, Georgeson L, Grace D, Graham H, Gunther SH, Hartinger S, He K, Heaviside C, Hess J, Hsu S-C, Jankin S, Jimenez MP, Kelman I, Kiesewetter G, Kinney PL, Kjellstrom T, Kniveton D, Lee JKW, Lemke B, Liu Y, Liu Z, Lott M, Lowe R, Martinez-Urtaza J, Maslin M, McAllister L, McMichael C, Mi Z, Milner J, Minor K, Mohajeri N, Moradi-Lakeh M, Morrissey K, Munzert S, Murray KA, Neville T, Nilsson M, Obradovich N, Sewe MO, Oreszczyn T, Otto M, Owfi F, Pearman O, Pencheon D, Rabbaniha M, Robinson E, Rocklöv J, Salas RN, Semenza JC, Sherman J, Shi L, Springmann M, Tabatabaei M, Taylor J, Trinanes J, Shumake-Guillemot J, Vu B, Wagner F, Wilkinson P, Winning M, Yglesias M, Zhang S, Gong P, Montgomery H, Costello A, Hamilton I. The 2021 report of the Lancet Countdown on health and climate change: code red for a healthy future. *Lancet*. 2021;398(10311):1619–62. 10.1016/S0140-6736(21)01787-6. 34687662 10.1016/S0140-6736(21)01787-6 PMC7616807

[55] Ruiz-Páez R, Díaz J, López-Bueno JA, Asensio C, Ascaso MS, Saez M, Barceló MA, Navas MA, Linares C. Short–term effects of air pollution and noise on emergency hospital admissions in Madrid and economic assessment. *Environ Res*. 2023;219: 115147. 36580986 10.1016/j.envres.2022.115147

[56] Ruiz-Páez R, Díaz J, López-Bueno JA, SaezM BMA, Navas MA, Linares C. Economic estimation and impact of air pollution and temperature extremes on emergency hospital admissions in Spain. *Sci Total Environ*. 2025;968: 178867. 39987822 10.1016/j.scitotenv.2025.178867

[57] Soares J, Plass D, Kienzler S, González Ortiz A, Gsella A, Horálek J. Health risk assessment of air pollution: assessing the environmental burden of disease in Europe in 2021 (Eionet Report – ETC HE 2023/7). *Eur Topic Centre Human Health Environ*. 2023. https://www.eionet.europa.eu/etcs/all-etc-reports. Accessed Feb 2025.

[58] Shin HH, Maquiling A, Thomson EM, Park IW, Stieb DM, Dehghani P. Sex-difference in air pollution-related acute circulatory and respiratory mortality and hospitalization. *Sci Total Environ*. 2022;806(Part 3):150515. 10.1016/j.scitotenv.2021.150515 34627116

[59] Stafoggia M, Samoli E, Alessandrini E, Cadum E, Ostro B, Berti G, Faustini A, Linares, members del MED-Particles Study Group. Short-term association between fine and coarse-particulate matter and hospitalizations in Southern Europe. Results from the MED-PARTICLES project. *Environ Health Perspect*. 2013;121:1026–33. 10.1289/ehp.1206151 PMC3764077 23777832

[60] Stafoggia M, Oftedal B, Chen J, Rodopoulou S, Renzi M, Atkinson RW, et al. Long-term exposure to low ambient air pollution concentrations and mortality among 28 million people: results from seven large European cohorts within the ELAPSE project. *Lancet Planet Health*. 2022;6(1):e9-18. 34998464 10.1016/S2542-5196(21)00277-1

[61] Stafoggia M, Michelozzi P, Schneider A, et al. Joint effect of heat and air pollution on mortality in 620 cities of 36 countries. *Environ Int*. 2023;181: 108258. 10.1016/J.ENVINT.2023.108258. 37837748 10.1016/j.envint.2023.108258 PMC10702017

[62] Thakur CP, Anand MP, Shahi MP. Cold weather and myocardial infarction. *Int J Cardiol*. 1987;16:19–25. 3610394 10.1016/0167-5273(87)90266-x

[63] United Nations. El cambio climático es un asunto de justicia: he aquí por qué. 2023. https://climatepromise.undp.org/es/news-and-stories/el-cambio-climatico-es-un-asunto-de-justicia-he-aqui-por-que. Accessed Feb 2025.

[64] Vodonos A, Friger M, Katra I, Krasnov H, Zahger D, Schwartz J, Novack V. Individual effect modifiers of dust exposure effect on cardiovascular morbidity. *PLoS One*. 2015;10(9):e0137714. 10.1371/journal.pone.0137714 PMC4575174 26381397

[65] Yu J, Zhu A, Liu M, Dong J, Chen R, et al. Association between air pollution and cardiovascular disease hospitalizations in Lanzhou City, 2013–2020: a time series analysis. *Geohealth*. 2024;8(1): e2022GH000780. 38173697 10.1029/2022GH000780 PMC10762694

[66] Wang L, Yang X, Dong J, et al. Evolution of surface ozone pollution pattern in eastern China and its relationship with different intensity heatwaves. *Environ Pollut*. 2023;338: 122725. 10.1016/J.ENVPOL.2023.122725. 37827354 10.1016/j.envpol.2023.122725

[67] WHO. *Review of evidence on health aspects of air pollution – REVIHAAP Project Technical Report*. Copenhagen: WHO Regional Office for Europe; 2013. 27195369

[68] WHO. Review of evidence on health aspects of air pollution: REVIHAAP project: technical report. 2021. https://www.who.int/europe/publications/i/item/WHO-EURO-2013-4101-43860-61757. Accessed Feb 2025. 27195369

[69] WHO. Health topics -* Air pollution*. 2024. Available from: https://www.who.int/health-topics/air-pollution#tab=tab_1. Accessed 20 Mar 2024.

