# Supplementary material for: Emergency Hospital Admissions for Cardiovascular Causes Attributable to Air Pollution and Extreme Temperatures in Spain: Influence of Economic and Demographic Factors in a Nationwide Study
Source: J Urban Health. 2025 Sep 2;102(4):813–29. doi: 10.1007/s11524-025-01006-6 (PMC12484484; doi:10.1007/s11524-025-01006-6)
Supplement: Supplementary file 1 — Supplementary Material 1 (DOCX 238 KB) [file 11524_2025_1006_MOESM1_ESM.docx]

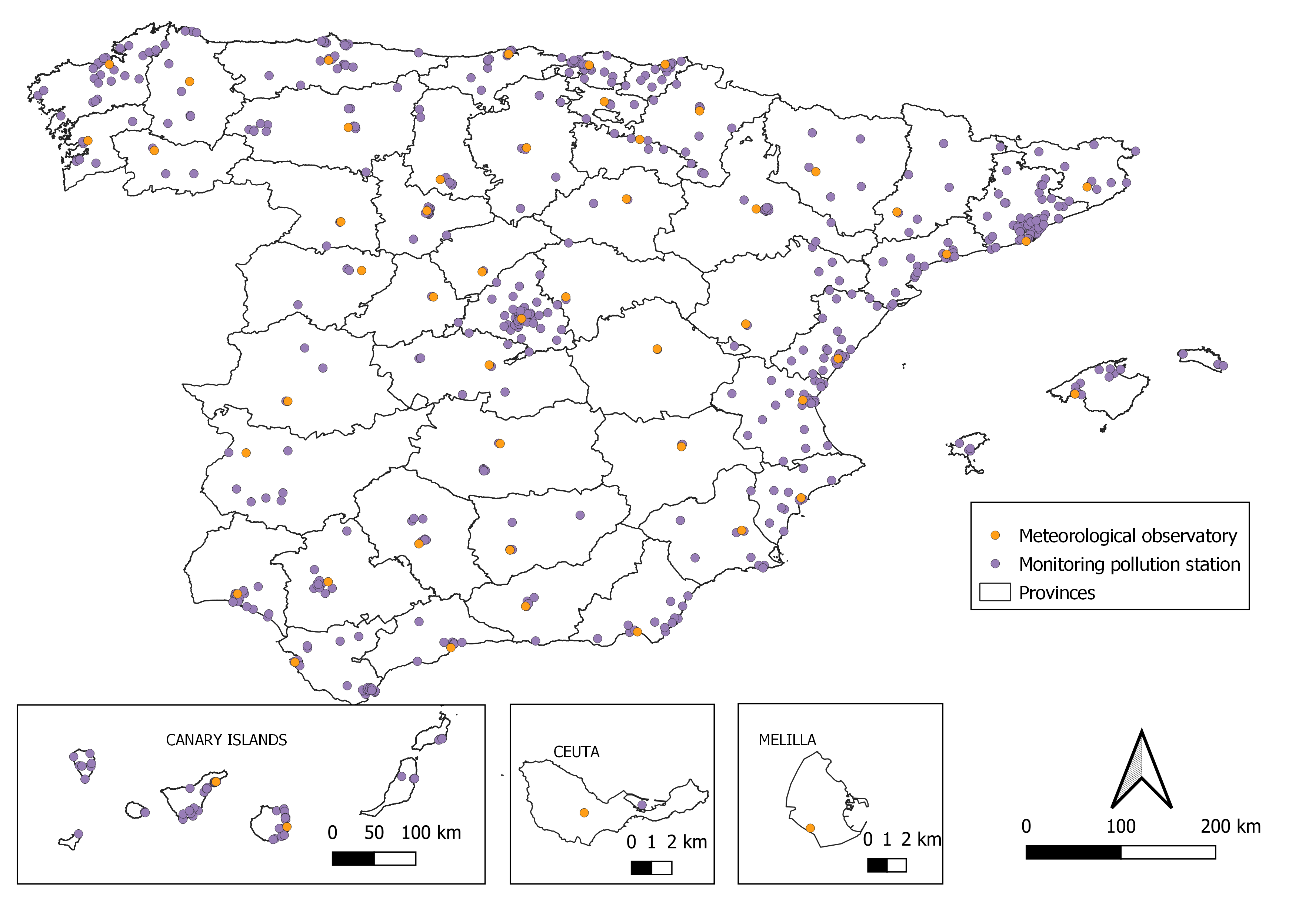


Figure S1. Situation of the respective Spanish provinces, along with the air pollution monitoring stations and meteorological observatories used in the study.

Table S1. Descriptive statistics of the dependent variables used in the study at a provincial level: 2013-2018

CVD: all cardiovascular disease; ACVA: acute cerebrovascular disease; AMI: acute myocardial infarction; IHD: ischaemic heart disease

| **Autonomous Region** | **Province** | **CVD** | | | **ACVA** | | | **AMI** | | | **IHD** | | |
| --- | --- | --- | --- | --- | --- | --- | --- | --- | --- | --- | --- | --- | --- |
|  |  | **Mean** | **SD** | **N** | **Mean** | **SD** | **N** | **Mean** | **SD** | **N** | **Mean** | **SD** | **N** |
| **Andalusia** | **Almeria** | 13.96 | 4.57 | 2191 | 2.61 | 1.64 | 2191 | 2.05 | 1.48 | 2191 | 0.04 | 0.19 | 2191 |
|  | **Cadiz** | 29.02 | 7.58 | 2191 | 4.55 | 2.18 | 2191 | 3.3 | 1.94 | 2191 | 0.26 | 0.53 | 2191 |
|  | **Cordoba** | 16.53 | 4.92 | 2191 | 3.28 | 1.87 | 2191 | 1.63 | 1.29 | 2191 | 0.02 | 0.14 | 2191 |
|  | **Granada** | 17.91 | 5.82 | 2191 | 3.52 | 1.99 | 2191 | 2.73 | 1.71 | 2191 | 0.03 | 0.18 | 2191 |
|  | **Huelva** | 11.55 | 5.42 | 2191 | 1.74 | 1.43 | 2191 | 1.1 | 1.12 | 2191 | 0.08 | 0.28 | 2191 |
|  | **Jaen** | 12.82 | 4.52 | 2191 | 2.64 | 1.67 | 2191 | 1.54 | 1.25 | 2191 | 0.02 | 0.14 | 2191 |
|  | **Malaga** | 30.93 | 7.84 | 2191 | 5.3 | 2.36 | 2191 | 4.67 | 2.18 | 2191 | 0.17 | 0.42 | 2191 |
|  | **Seville** | 40.06 | 11.1 | 2191 | 6.4 | 2.67 | 2191 | 5.43 | 2.57 | 2191 | 0.26 | 0.56 | 2191 |
| **Aragon** | **Huesca** | 5.97 | 2.77 | 2191 | 0.99 | 1.02 | 2191 | 0.3 | 0.56 | 2191 | 0.01 | 0.09 | 2191 |
|  | **Teruel** | 3.2 | 1.9 | 2191 | 0.51 | 0.71 | 2191 | 0.14 | 0.38 | 2191 | 0.01 | 0.08 | 2191 |
|  | **Zaragoza** | 24.23 | 7.31 | 2191 | 3.99 | 2.06 | 2191 | 2.28 | 1.54 | 2191 | 0.07 | 0.26 | 2191 |
| **Asturias** | **Asturias** | 35.05 | 9.51 | 2191 | 6.23 | 2.58 | 2191 | 3.36 | 1.87 | 2191 | 0.12 | 0.37 | 2191 |
| **Balearic Isles** | **Balearics** | 22.87 | 7.13 | 2191 | 3.33 | 1.95 | 2191 | 2.42 | 1.59 | 2191 | 0.1 | 0.33 | 2191 |
| **Basque Country** | **Alava** | 8.06 | 3.17 | 2191 | 1.35 | 1.15 | 2191 | 0.76 | 0.86 | 2191 | 0.01 | 0.11 | 2191 |
|  | **Bizkaia** | 32.84 | 8.82 | 2191 | 4.86 | 2.3 | 2191 | 2.59 | 1.69 | 2191 | 0.11 | 0.34 | 2191 |
|  | **Gipuzkoa** | 16.53 | 5.19 | 2191 | 3.12 | 1.78 | 2191 | 1.44 | 1.23 | 2191 | 0.08 | 0.28 | 2191 |
| **Canary Islands** | **Las Palmas** | 18.91 | 5.81 | 2191 | 3.19 | 1.84 | 2191 | 3.51 | 1.91 | 2191 | 0.11 | 0.35 | 2191 |
|  | **SC Tfe** | 17.94 | 6.01 | 2191 | 2.88 | 1.7 | 2191 | 2.14 | 1.48 | 2191 | 0.05 | 0.25 | 2191 |
| **Cantabria** | **Cantabria** | 15.97 | 5.06 | 2191 | 2.68 | 1.68 | 2191 | 1.53 | 1.27 | 2191 | 0.06 | 0.26 | 2191 |
| **Castile & Leon** | **Avila** | 4.76 | 2.45 | 2191 | 0.77 | 0.89 | 2191 | 0.33 | 0.6 | 2191 | 0.01 | 0.11 | 2191 |
|  | **Burgos** | 10.46 | 4.68 | 2191 | 1.64 | 1.34 | 2191 | 0.77 | 0.93 | 2191 | 0.02 | 0.14 | 2191 |
|  | **Leon** | 14.23 | 5.6 | 2191 | 2.02 | 1.53 | 2191 | 1.66 | 1.31 | 2191 | 0.06 | 0.24 | 2191 |
|  | **Palencia** | 5.09 | 2.64 | 2191 | 0.7 | 0.85 | 2191 | 0.47 | 0.69 | 2191 | 0 | 0.06 | 2191 |
|  | **Salamanca** | 9.22 | 3.37 | 2191 | 1.5 | 1.26 | 2191 | 1.14 | 1.06 | 2191 | 0.01 | 0.11 | 2191 |
|  | **Segovia** | 3.93 | 2.1 | 2191 | 0.72 | 0.84 | 2191 | 0.44 | 0.65 | 2191 | 0 | 0.06 | 2191 |
|  | **Soria** | 2.85 | 1.89 | 2191 | 0.45 | 0.68 | 2191 | 0.27 | 0.53 | 2191 | 0.01 | 0.09 | 2191 |
|  | **Valladolid** | 12.51 | 4.88 | 2191 | 1.84 | 1.41 | 2191 | 1.48 | 1.27 | 2191 | 0.07 | 0.27 | 2191 |
|  | **Zamora** | 5.67 | 2.55 | 2191 | 0.98 | 0.98 | 2191 | 0.56 | 0.76 | 2191 | 0 | 0.04 | 2191 |
| **Castile-La Mancha** | **Albacete** | 9.64 | 3.48 | 2191 | 1.83 | 1.36 | 2191 | 1.03 | 1 | 2191 | 0.02 | 0.14 | 2191 |
|  | **Ciudad Real** | 12.55 | 4.88 | 2191 | 2.18 | 1.56 | 2191 | 1.35 | 1.21 | 2191 | 0.02 | 0.15 | 2191 |
|  | **Cuenca** | 4.17 | 2.19 | 2191 | 0.83 | 0.92 | 2191 | 0.23 | 0.48 | 2191 | 0 | 0.04 | 2191 |
|  | **Guadalajara** | 5.12 | 2.56 | 2191 | 0.69 | 0.84 | 2137 | 0.45 | 0.68 | 2137 | 0 | 0.05 | 2137 |
|  | **Toledo** | 9.48 | 5.75 | 2191 | 1.69 | 1.59 | 2191 | 1.11 | 1.19 | 2191 | 0.02 | 0.15 | 2191 |
| **Catalonia** | **Barcelona** | 146.79 | 30.35 | 2191 | 23.61 | 5.53 | 2191 | 14.85 | 4.38 | 2191 | 0.28 | 0.56 | 2191 |
|  | **Gerona** | 14.57 | 4.57 | 2190 | 2.43 | 1.54 | 2190 | 2 | 1.4 | 2190 | 0.05 | 0.22 | 2190 |
|  | **Lleida** | 7.54 | 3.02 | 2191 | 1.31 | 1.14 | 2191 | 0.77 | 0.9 | 2191 | 0.04 | 0.21 | 2191 |
|  | **Tarragona** | 16.51 | 4.87 | 2191 | 2.69 | 1.67 | 2191 | 2.32 | 1.51 | 2191 | 0.05 | 0.21 | 2191 |
| **Ceuta** | **Ceuta** | 1.43 | 1.24 | 2191 | 0.26 | 0.52 | 2191 | 0.16 | 0.4 | 2191 | 0.01 | 0.09 | 2191 |
| **Extremadura** | **Badajoz** | 19.03 | 5.78 | 2191 | 2.72 | 1.72 | 2191 | 1.84 | 1.36 | 2191 | 0.12 | 0.35 | 2191 |
|  | **Cáceres** | 10.58 | 3.83 | 2191 | 2.03 | 1.47 | 2191 | 1.41 | 1.21 | 2191 | 0.04 | 0.2 | 2191 |
| **Galicia** | **A Coruña (Corunna)** | 31.87 | 8.13 | 2191 | 5 | 2.36 | 2191 | 3.72 | 1.96 | 2191 | 0.07 | 0.27 | 2191 |
|  | **Lugo** | 8.71 | 3.98 | 2191 | 1.66 | 1.35 | 2191 | 0.73 | 0.87 | 2191 | 0.02 | 0.14 | 2191 |
|  | **Ourense** | 12.25 | 3.99 | 2191 | 2.29 | 1.56 | 2191 | 0.81 | 0.9 | 2191 | 0.02 | 0.13 | 2191 |
|  | **Pontevedra** | 27.11 | 7.16 | 2191 | 4.46 | 2.21 | 2191 | 2.68 | 1.66 | 2191 | 0.07 | 0.26 | 2191 |
| **La Rioja** | **La Rioja** | 9.1 | 3.54 | 2191 | 1.49 | 1.24 | 2191 | 1.03 | 1.01 | 2191 | 0.01 | 0.09 | 2191 |
| **Madrid** | **Madrid** | 149.01 | 33.69 | 2191 | 23.16 | 5.74 | 2191 | 14.06 | 4.22 | 2191 | 0.35 | 0.62 | 2191 |
| **Melilla** | **Melilla** | 1.27 | 1.16 | 2191 | 0.23 | 0.48 | 2191 | 0.2 | 0.45 | 2191 | 0.03 | 0.18 | 2191 |
| **Murcia** | **Murcia** | 40.97 | 9.96 | 2191 | 6.01 | 2.58 | 2191 | 4.52 | 2.23 | 2191 | 0.23 | 0.48 | 2191 |
| **Navarre** | **Navarre** | 14.01 | 4.28 | 2191 | 2.65 | 1.65 | 2191 | 1.52 | 1.24 | 2191 | 0.05 | 0.21 | 2191 |
| **Valencia** | **Alicante** | 52.79 | 13.41 | 2191 | 8.42 | 3.18 | 2191 | 5.45 | 2.41 | 2191 | 0.14 | 0.38 | 2191 |
|  | **Castellón** | 11.86 | 4.81 | 2191 | 2.32 | 1.59 | 2191 | 1.09 | 1.07 | 2191 | 0.07 | 0.26 | 2191 |
|  | **Valencia** | 57.88 | 13.05 | 2191 | 10.35 | 3.48 | 2191 | 6.24 | 2.66 | 2191 | 0.57 | 0.78 | 2191 |

Table S2. Descriptive statistics of the independent variables used in the study at a provincial level (2013-2018). n: number of days with heat wave (Theat) or cold wave (Tcold)

| **Autonomous Region** | **Province** | **NO_2_ (µg/m3)** | **O_3_ (µg/m3)** | **PM_10_ (µg/m3)** | **PM_2.5_ (µg/m3)** | **Tmax (⁰C)** | **Tmin (⁰C)** | **Theat (⁰C)** |  | **Tcold (⁰C)** |  |
| --- | --- | --- | --- | --- | --- | --- | --- | --- | --- | --- | --- |
| **Mean ± sd** | **Mean ± sd** | **Mean ± sd** | **Mean ± sd** | **Mean ± sd** | **Mean ± sd** | **Mean ± sd** | **Mean ± sd** | **Mean ± sd** | **n** | **Mean ± sd** | **n** |
| **Andalusia** | **Almeria** | 14.9 ± 5.0 | 73.3 ± 14.8 | 25.0 ± 11.6 | - | 23.8 ± 5.8 | 15.4 ± 5.5 | 1.5 ± 1.3 | 69 | 0.7 ± 0.7 | 31 |
|  | **Cadiz** | 13.6 ± 5.8 | 68.6 ± 17.6 | 24.8 ± 10.8 | - | 22.0 ± 5.1 | 16.1 ± 4.9 | - | 0 | - | 0 |
|  | **Cordoba** | 23.6 ± 9.1 | 58.6 ± 22.9 | - | - | 26.2 ± 8.9 | 11.3 ± 6.5 | 1.7 ± 1.3 | 48 | 1.5 ± 1.1 | 164 |
|  | **Granada** | 26.5 ± 10.3 | 63.5 ± 18.2 | 26.4 ± 15.0 | 13.9 ± 8.5 | 23.3 ± 8.6 | 9.0 ± 6.8 | 1.7 ± 1.4 | 122 | 1.5 ± 1.1 | 176 |
|  | **Huelva** | 7.8 ± 4.1 | 66.8 ± 17.2 | 20.8 ± 11.5 | - | 24.5 ± 6.7 | 12.8 ± 5.4 | 1.2 ± 1.1 | 34 | 1.7 ± 1.2 | 163 |
|  | **Jaén** | 17.9 ± 8.9 | 71.5 ± 22.9 | - | - | 22.7 ± 8.9 | 12.5 ± 6.5 | 1.4 ± 1.1 | 31 | 1.0 ± 0.9 | 40 |
|  | **Malaga** | 24.8 ± 9.3 | 64.9 ± 17.2 | - | - | 24.1 ± 5.8 | 14.8 ± 5.6 | 1.6 ± 1.1 | 32 | 0.9 ± 0.8 | 21 |
|  | **Seville** | 20.2 ± 7.5 | 57.2 ± 18.7 | 25.7 ± 13.2 | - | 25.9 ± 8.0 | 13.3 ± 5.9 | 1.2 ± 1.1 | 54 | 1.3 ± 1.0 | 157 |
| **Aragon** | **Huesca** | 13.1 ± 6.3 | 62.2 ± 20.3 | 15.0 ± 8.1 | - | 20.6 ± 8.7 | 9.0 ± 6.3 | 1.4 ± 1.3 | 112 | 1.6 ± 1.3 | 90 |
|  | **Teruel** | 8.7 ± 3.7 | 65.8 ± 18.9 | 12.2 ± 8.5 | - | 20.7 ± 8.8 | 5.9 ± 6.6 | 0.8 ± 0.7 | 38 | 1.1 ± 1.0 | 32 |
|  | **Zaragoza** | 24.1 ± 8.5 | 51.1 ± 20.8 | 18.4 ± 9.5 | - | 22.0 ± 8.7 | 10.9 ± 6.4 | 1.3 ± 1.3 | 28 | 1.5 ± 1.3 | 85 |
| **Asturias** | **Asturias** | 19.8 ± 7.8 | 43.9 ± 14.9 | 23.5 ± 7.3 | 10.2 ± 4.8 | 17.7 ± 5.6 | 9.5 ± 4.6 | 2.1 ± 1.9 | 145 | 0.9 ± 0.9 | 11 |
| **Balearic Isles** | **Balearics** | 9.5 ± 3.1 | 68.1 ± 14.0 | 17.2 ± 6.3 | 6.9 ± 3.0 | 22.7 ± 5.8 | 15.2 ± 5.7 | 1.3 ± 1.3 | 34 | 1.2 ± 1.0 | 52 |
| **Basque Country** | **Araba** | 20.1 ± 9.4 | 65.4 ± 17.7 | 14.0 ± 7.5 | - | 18.0 ± 7.7 | 6.6 ± 5.3 | 1.8 ± 1.2 | 64 | 1.6 ± 1.2 | 24 |
|  | **Bizkaia** | 24.2 ± 9.2 | 49.9 ± 16.5 | 17.6 ± 7.8 | 10.6 ± 5.6 | 20.0 ± 6.3 | 10.4 ± 5.1 | 2.9 ± 2.0 | 45 | 1.4 ± 1.2 | 40 |
|  | **Gipuzkoa** | 21.9 ± 8.3 | 52.1 ± 16.8 | 15.8 ± 6.2 | 8.5 ± 4.6 | 17.0 ± 5.7 | 11.0 ± 4.8 | 3.4 ± 2.7 | 74 | 1.8 ± 1.4 | 41 |
| **Canary Islands** | **Las Palmas** | 12.6 ± 4.8 | 61.5 ± 13.5 | 27.2 ± 23.6 | 8.6 ± 6.1 | 24.7 ± 3.1 | 18.7 ± 2.8 | 1.4 ± 1.2 | 25 | 1.5 ± 2.5 | 27 |
|  | **S.C. de Tenerife** | 11.1 ± 4.7 | 61.9 ± 13.9 | 22.1 ± 22.0 | 9.1 ± 7.0 | 25.0 ± 3.5 | 18.8 ± 2.8 | 1.3 ± 1.0 | 16 | 0.7 ± 0.6 | 32 |
| **Cantabria** | **Cantabria** | 17.6 ± 6.7 | 55.2 ± 16.3 | 18.8 ± 7.4 | - | 19.0 ± 5.0 | 11.4 ± 4.7 | 2.3 ± 1.9 | 112 | 1.3 ± 0.8 | 13 |
| **Castile & Leon** | **Avila** | 9.2 ± 6.3 | 62.0 ± 19.7 | 16.1 ± 9.2 | - | 17.8 ± 8.7 | 6.4 ± 6.3 | 1.1 ± 0.9 | 59 | 1.6 ± 1.5 | 62 |
|  | **Burgos** | 11.2 ± 6.4 | 59.4 ± 16.5 | 15.8 ± 9.4 | 8.1 ± 5.1 | 17.1 ± 8.6 | 5.2 ± 5.5 | 1.2 ± 0.8 | 43 | 1.8 ± 1.5 | 111 |
|  | **Leon** | 15.2 ± 7.6 | 55.6 ± 16.3 | 16.0 ± 7.9 | - | 17.3 ± 8.2 | 5.5 ± 5.7 | 1.1 ± 0.7 | 26 | 0.8 ± 0.9 | 16 |
|  | **Palencia** | 10.8 ± 9.2 | 60.5 ± 21.5 | 18.5 ± 11.5 | - | 17.5 ± 8.7 | 5.6 ± 5.7 | 1.5 ± 1.1 | 68 | 0.9 ± 0.5 | 20 |
|  | **Salamanca** | 11.0 ± 6.3 | 68.9 ± 16.7 | 17.0 ± 9.4 | - | 19.9 ± 8.8 | 5.2 ± 5.9 | 1.5 ± 1.0 | 69 | 2.0 ± 1.6 | 200 |
|  | **Segovia** | 12.6 ± 6.5 | 64.8 ± 20.2 | 13.8 ± 11.1 | - | 18.4 ± 8.9 | 7.4 ± 6.3 | 1.2 ± 0.9 | 80 | 1.7 ± 1.5 | 113 |
|  | **Soria** | 12.7 ± 6.8 | 59.7 ± 17.8 | 15.2 ± 10.6 | - | 178.5 ± 86.2 | 5.3 ± 5.8 | 0.9 ± 0.7 | 26 | 1.2 ± 1.0 | 31 |
|  | **Valladolid** | 23.2 ± 10.6 | 52.1 ± 21.9 | 16.1 ± 9.2 | 11.2 ± 6.3 | 19.3 ± 9.1 | 7.3 ± 6.0 | 1.1 ± 0.7 | 44 | 1.4 ± 1.0 | 133 |
|  | **Zamora** | 5.1 ± 3.4 | 67.1 ± 16.8 | 12.1 ± 7.6 | 4.7 ± 3.5 | 20.1 ± 9.2 | 7.8 ± 6.1 | 1.1 ± 0.9 | 42 | 1.7 ± 1.3 | 163 |
| **Castile-La Mancha** | **Albacete** | - | - | - | - | 21.7 ± 8.8 | 8.6 ± 7.0 | 1.2 ± 0.9 | 36 | 1.7 ± 1.4 | 129 |
|  | **Ciudad Real** | 10.4 ± 7.9 | 61.6 ± 21.3 | - | - | 22.5 ± 9.3 | 10.1 ± 7.1 | 1.4 ± 1.0 | 72 | 1.7 ± 1.2 | 151 |
|  | **Cuenca** | 16.8 ± 11.4 | - | - | - | 20.7 ± 8.9 | 7.8 ± 6.8 | 1.1 ± 0.9 | 50 | 1.6 ± 1.1 | 88 |
|  | **Guadalajara** | 10.1 ± 5.9 | 63.0 ± 17.9 | 15.7 ± 12.4 | - | - | - | - | - | - | - |
|  | **Toledo** | 15.4 ± 7.8 | 63.7 ± 20.8 | 21.3 ± 11.4 | 8.6 ± 4.0 | 23.1 ± 9.2 | 10.2 ± 7.1 | 1.7 ± 1.2 | 98 | 1.4 ± 1.1 | 96 |
| **Catalonia** | **Barcelona** | 30.5 ± 10.0 | 48.8 ± 19.3 | 23.1 ± 7.9 | 14.2 ± 4.9 | 21.3 ± 5.7 | 13.6 ± 6.3 | 1.3 ± 1.3 | 50 | 1.2 ± 0.9 | 79 |
|  | **Girona** | 15.5 ± 6.8 | 70 ± 14.9 | 19.9 ± 7.1 | 9.2 ± 5.1 | 22.4 ± 7.1 | 8.9 ± 6.6 | 1.5 ± 1.4 | 130 | 1.8 ± 1.4 | 203 |
|  | **Lleida** | 9.0 ± 4.4 | 62.5 ± 19.7 | 15.4 ± 7.8 | 9.4 ± 5.5 | 22.2 ± 9.1 | 9.4 ± 6.9 | 1.2 ± 1.0 | 36 | 1.0 ± 0.9 | 48 |
|  | **Tarragona** | 14.2 ± 6.4 | 66.5 ± 18.0 | 16.7 ± 7.8 | - | 22.7 ± 6.2 | 11.6 ± 6.7 | 1.7 ± 1.3 | 8 | 1.6 ± 1.1 | 72 |
| **Ceuta** | **Ceuta** | - | - | - | - | 21.9 ± 5.1 | 16.0 ± 4.1 | 2.0 ± 1.7 | 45 | 1.1 ± 0.8 | 43 |
| **Extremadura** | **Badajoz** | 7.2 ± 4.2 | 60.2 ± 17.4 | 15.2 ± 9.9 | 8.6 ± 4.5 | 24.5 ± 8.4 | 11.1 ± 6.0 | 1.5 ± 1.3 | 20 | 1.8 ± 1.2 | 112 |
|  | **Cáceres** | 6.5 ± 3.4 | 65.1 ± 21.6 | 11.2 ± 7.6 | 6.3 ± 4.1 | 22.5 ± 8.8 | 11.0 ± 6.3 | 1.8 ± 1.4 | 104 | 1.4 ± 1.1 | 77 |
| **Navarre** | **Navarre** | 18.6 ± 9.4 | 52.2 ± 19.3 | 15.4 ± 6.5 | - | 19.3 ± 8.4 | 7.6 ± 5.7 | 1.7 ± 1.2 | 105 | 1.0 ± 0.9 | 23 |
| **Galicia** | **A Coruña (Corunna)** | 15.9 ± 7.4 | 54.8 ± 15.5 | 22.6 ± 8.9 | 12.6 ± 6.8 | 18.9 ± 5.0 | 10.1 ± 4.5 | 2.1 ± 4.0 | 132 | 1.6 ± 1.1 | 105 |
|  | **Lugo** | 6.1 ± 3.5 | 54.2 ± 15.6 | 12.1 ± 6.1 | 8.5 ± 4.7 | 18.1 ± 6.9 | 7.2 ± 5.0 | 1.9 ± 1.5 | 41 | 0.7 ± 0.7 | 8 |
|  | **Ourense** | 12.6 ± 5.7 | 50.0 ± 17.4 | 13.8 ± 9.7 | - | 22.2 ± 7.9 | 9.1 ± 5.4 | 1.7 ± 1.3 | 47 | 2.1 ± 1.6 | 189 |
|  | **Pontevedra** | 20.8 ± 10.2 | 46.2 ± 18.1 | 20.7 ± 9.5 | - | 19.1 ± 6.0 | 10.7 ± 4.3 | 2.7 ± 2.2 | 157 | 1.6 ± 1.1 | 220 |
| **La Rioja** | **La Rioja** | 13.8 ± 9.5 | 48.2 ± 19.5 | 19.9 ± 6.7 | - | 20.3 ± 8.3 | 8.6 ± 5.7 | 1.3 ± 1.0 | 92 | 1.2 ± 1.0 | 37 |
| **Madrid** | **Madrid** | 30.7 ± 14.5 | 56.4 ± 23.0 | 19 ± 9.7 | 10.3 ± 4.7 | 21.1 ± 9.1 | 11.1 ± 6.8 | 2.0 ± 1.5 | 232 | 0.7 ± 0.3 | 4 |
| **Melilla** | **Melilla** | - | - | - | - | 22.6 ± 5.0 | 16.2 ± 4.9 | 1.8 ± 1.1 | 29 | 1.0 ± 0.9 | 63 |
| **Murcia** | **Murcia** | 16.9 ± 7.0 | 67.3 ± 17.3 | 20.6 ± 10.6 | - | 26.1 ± 6.9 | 13.6 ± 6.3 | 1.6 ± 1.4 | 32 | 1.5 ± 1.1 | 79 |
| **Valencia** | **Alicante** | 14.5 ± 6.9 | 65.3 ± 16.4 | 17.1 ± 8.5 | 10.4 ± 4.9 | 23.8 ± 5.6 | 13.9 ± 5.9 | 1.7 ± 1.6 | 119 | 1.4 ± 1.0 | 82 |
|  | **Castellón** | 8.7 ± 3.8 | 64.0 ± 15.3 | 13.2 ± 7.4 | 8.5 ± 4.7 | 23.1 ± 5.9 | 13.9 ± 5.8 | 1.0 ± 0.9 | 75 | 1.2 ± 0.8 | 56 |
|  | **Valencia** | 16.7 ± 6.9 | 61.8 ± 16.4 | 17.3 ± 7.8 | 9.4 ± 4.7 | 23.7 ± 6.1 | 12.6 ± 6.5 | 2.0 ± 1.7 | 36 | 1.6 ± 1.3 | 148 |

Table S3. Relative risks (RR) and attributable risks (AR), with their confidence intervals at a provincial level for each of the causes of admission, along with the factor shown to be statistically significant in the Poisson modelling.

CVD: all cardiovascular disease; ACVA: acute cerebrovascular disease; AMI: acute myocardial infarction; IHD: ischaemic heart disease

| **Province** | **Cause** | **Risk Factor** | **RR** | **Lower** | **Upper** | **AR%** | **Lower** | **Upper** |
| --- | --- | --- | --- | --- | --- | --- | --- | --- |
| A Coruña (Corunna) | CVD | Tcold | 1.05 | 1.01 | 1.09 | 4.59 | 1.2 | 7.86 |
| A Coruña | ACVA | Tcold | 1.05 | 1.01 | 1.10 | 4.92 | 0.94 | 8.73 |
| A Coruña | IHD | O3 | 1.12 | 1.01 | 1.24 | 10.72 | 1.29 | 19.25 |
| Álava | CVD | Tcold | 1.10 | 1.03 | 1.17 | 8.81 | 2.93 | 14.35 |
| Álava | ACVA | Tcold | 1.19 | 1.03 | 1.36 | 15.75 | 3.33 | 26.57 |
| Álava | IHD | Tcold | 2.25 | 1.17 | 4.34 | 55.62 | 14.54 | 76.96 |
| Álava | AMI | NO2 | 1.07 | 1.01 | 1.12 | 6.12 | 1.1 | 10.88 |
| Albacete | CVD | Tcold | 1.03 | 1.00 | 1.05 | 2.74 | 0.25 | 5.17 |
| Alicante | CVD | O3 | 1.01 | 1.01 | 1.02 | 1.32 | 0.73 | 1.92 |
| Alicante | CVD | Tcold | 1.04 | 1.01 | 1.08 | 4 | 0.69 | 7.2 |
| Alicante | ACVA | Tcold | 1.06 | 1.01 | 1.10 | 5.25 | 1.35 | 8.98 |
| Alicante | IHD | PM10 | 1.17 | 1.04 | 1.31 | 14.41 | 4.28 | 23.46 |
| Alicante | AMI | O3 | 1.03 | 1.01 | 1.05 | 2.89 | 1.33 | 4.43 |
| Alicante | AMI | Theat | 1.04 | 1.01 | 1.07 | 3.82 | 0.56 | 6.97 |
| Almería | ACVA | Tcold | 1.23 | 1.03 | 1.47 | 18.56 | 2.77 | 31.78 |
| Asturias | CVD | O3 | 1.01 | 1.01 | 1.02 | 1.2 | 0.51 | 1.89 |
| Asturias | CVD | NO2 | 1.02 | 1.01 | 1.04 | 2.15 | 0.65 | 3.63 |
| Asturias | CVD | PM10 | 1.01 | 1.00 | 1.01 | 1.34 | 0.35 | 0.53 |
| Asturias | CVD | Tcold | 1.32 | 1.24 | 1.42 | 24.51 | 19.04 | 29.61 |
| Asturias | ACVA | O3 | 1.03 | 1.01 | 1.04 | 2.57 | 1.31 | 3.82 |
| Asturias | ACVA | Tcold | 1.18 | 1.01 | 1.37 | 14.9 | 0.95 | 26.89 |
| Asturias | IHD | O3 | 1.43 | 1.27 | 1.61 | 29.99 | 21.15 | 37.85 |
| Asturias | IHD | NO2 | 1.87 | 1.47 | 2.38 | 46.55 | 32.06 | 57.95 |
| Asturias | IHD | Tcold | 1.86 | 1.11 | 3.12 | 46.18 | 9.57 | 67.97 |
| Avila | CVD | Tcold | 1.06 | 1.01 | 1.11 | 5.45 | 0.83 | 9.85 |
| Avila | ACVA | Tcold | 1.27 | 1.04 | 1.56 | 21.45 | 3.45 | 36.1 |
| Avila | AMI | Tcold | 1.16 | 1.01 | 1.33 | 13.81 | 1.16 | 24.84 |
| Badajoz | CVD | PM2.5 | 1.04 | 1.01 | 1.06 | 3.48 | 1.21 | 5.7 |
| Badajoz | CVD | Tcold | 1.06 | 1.01 | 1.10 | 5.27 | 1.29 | 9.08 |
| Badajoz | ACVA | Tcold | 1.07 | 1.02 | 1.13 | 6.69 | 1.99 | 11.16 |
| Badajoz | AMI | Tcold | 1.09 | 1.03 | 1.15 | 8.31 | 2.96 | 13.37 |
| Balearics | CVD | PM10 | 1.02 | 1.01 | 1.03 | 1.87 | 0.5 | 3.22 |
| Balearics | CVD | Theat | 1.06 | 1.02 | 1.10 | 5.6 | 2.09 | 8.97 |
| Balearics | CVD | Tcold | 1.13 | 1.05 | 1.20 | 11.15 | 5.18 | 16.75 |
| Balearics | ACVA | PM2.5 | 1.08 | 1.00 | 1.17 | 7.76 | 0.39 | 14.59 |
| Balearics | ACVA | Theat | 1.16 | 1.07 | 1.25 | 13.5 | 6.54 | 19.94 |
| Balearics | ACVA | Tcold | 1.13 | 1.04 | 1.22 | 11.23 | 3.7 | 18.17 |
| Balearics | IHD | PM10 | 1.21 | 1.03 | 1.41 | 17.17 | 3.22 | 29.1 |
| Balearics | IHD | Theat | 3.03 | 1.60 | 5.76 | 67.03 | 37.42 | 82.63 |
| Balearics | IHD | Tcold | 1.56 | 1.05 | 2.31 | 35.81 | 4.92 | 56.66 |
| Balearics | AMI | Tcold | 1.11 | 1.00 | 1.22 | 9.68 | 0.13 | 18.33 |
| Barcelona | CVD | Tcold | 1.02 | 1.01 | 1.03 | 2 | 0.86 | 3.13 |
| Barcelona | IHD | PM2.5 | 1.24 | 1.06 | 1.44 | 19.15 | 5.93 | 30.5 |
| Barcelona | AMI | Tcold | 1.05 | 1.01 | 1.09 | 4.65 | 1.19 | 7.99 |
| Burgos | CVD | O3 | 1.01 | 1.00 | 1.02 | 1.4 | 0.37 | 2.42 |
| Burgos | CVD | NO2 | 1.06 | 1.03 | 1.08 | 5.44 | 3.21 | 7.61 |
| Burgos | CVD | Tcold | 1.03 | 1.01 | 1.05 | 2.87 | 0.55 | 5.14 |
| Burgos | ACVA | Tcold | 1.07 | 1.02 | 1.13 | 6.91 | 1.7 | 11.84 |
| Burgos | IHD | Tcold | 1.47 | 1.09 | 1.97 | 31.79 | 8.34 | 49.24 |
| Cáceres | CVD | PM2.5 | 1.04 | 1.01 | 1.07 | 3.87 | 0.79 | 6.86 |
| Cáceres | CVD | Tcold | 1.05 | 1.01 | 1.08 | 4.39 | 0.98 | 7.69 |
| Cáceres | ACVA | PM10 | 1.06 | 1.02 | 1.10 | 5.26 | 1.71 | 8.69 |
| Cadiz | CVD | NO2 | 1.04 | 1.01 | 1.07 | 3.81 | 0.87 | 6.66 |
| Cadiz | ACVA | O3 | 1.04 | 1.00 | 1.08 | 3.73 | 0.1 | 7.22 |
| Cadiz | IHD | NO2 | 1.25 | 1.08 | 1.44 | 20.06 | 7.75 | 30.73 |
| Cantabria | CVD | O3 | 1.02 | 1.00 | 1.03 | 1.59 | 0.21 | 2.95 |
| Cantabria | CVD | PM10 | 1.03 | 1.02 | 1.05 | 3.31 | 1.48 | 5.11 |
| Cantabria | ACVA | NO2 | 1.04 | 1.00 | 1.09 | 4.24 | 0.45 | 7.89 |
| Cantabria | ACVA | Tcold | 1.21 | 1.00 | 1.46 | 17.14 | 0.05 | 31.31 |
| Cantabria | IHD | NO2 | 1.29 | 1.02 | 1.64 | 22.54 | 1.75 | 38.93 |
| Cantabria | AMI | O3 | 1.04 | 1.01 | 1.07 | 3.71 | 0.91 | 6.44 |
| Castellón | CVD | Tcold | 1.14 | 1.04 | 1.26 | 12.44 | 3.48 | 20.58 |
| Castellón | ACVA | Tcold | 1.22 | 1.10 | 1.35 | 17.92 | 9 | 25.96 |
| Castellón | IHD | Theat | 1.68 | 1.22 | 2.33 | 40.53 | 17.75 | 57.01 |
| Ceuta | CVD | Theat | 1.10 | 1.01 | 1.19 | 8.94 | 1.35 | 15.94 |
| Ceuta | CVD | Tcold | 1.25 | 1.08 | 1.45 | 20.24 | 7.76 | 31.03 |
| Ceuta | ACVA | Theat | 1.21 | 1.05 | 1.41 | 17.68 | 4.72 | 28.88 |
| Ciudad Real | CVD | NO2 | 1.03 | 1.01 | 1.04 | 2.52 | 0.78 | 4.23 |
| Ciudad Real | CVD | Tcold | 1.04 | 1.02 | 1.06 | 3.99 | 1.93 | 6 |
| Ciudad Real | ACVA | O3 | 1.03 | 1.01 | 1.05 | 2.77 | 0.9 | 4.61 |
| Ciudad Real | ACVA | NO2 | 1.06 | 1.02 | 1.11 | 5.85 | 1.86 | 9.69 |
| Ciudad Real | ACVA | Tcold | 1.11 | 1.06 | 1.16 | 9.81 | 5.32 | 14.08 |
| Ciudad Real | IHD | Tcold | 1.56 | 1.14 | 2.15 | 36.05 | 12.05 | 53.51 |
| Ciudad Real | AMI | Tcold | 1.09 | 1.03 | 1.16 | 8.61 | 3.15 | 13.76 |
| Cordoba | CVD | Tcold | 1.04 | 1.02 | 1.06 | 3.57 | 1.62 | 5.48 |
| Cordoba | ACVA | Tcold | 1.16 | 1.06 | 1.26 | 13.5 | 5.7 | 20.65 |
| Cordoba | IHD | Theat | 1.82 | 1.04 | 3.19 | 45.17 | 4.04 | 68.67 |
| Cordoba | AMI | Theat | 1.11 | 1.00 | 1.22 | 9.77 | 0.31 | 18.33 |
| Cuenca | CVD | NO2 | 1.04 | 1.02 | 1.07 | 3.97 | 1.6 | 6.29 |
| Cuenca | CVD | Tcold | 1.05 | 1.00 | 1.11 | 5.2 | 0.39 | 9.79 |
| Cuenca | AMI | Theat | 1.39 | 1.05 | 1.85 | 28.31 | 4.75 | 46.05 |
| Gerona | CVD | O3 | 1.38 | 1.05 | 1.82 | 27.69 | 4.87 | 45.04 |
| Gerona | IHD | PM2.5 | 1.36 | 1.00 | 1.85 | 26.57 | 0.46 | 45.83 |
| Granada | CVD | Tcold | 1.02 | 1.00 | 1.04 | 1.96 | 0.15 | 3.74 |
| Granada | ACVA | Tcold | 1.04 | 1.00 | 1.09 | 4.28 | 0.32 | 8.07 |
| Granada | IHD | PM2.5 | 1.35 | 1.08 | 1.69 | 25.9 | 7.27 | 40.79 |
| Granada | IHD | Tcold | 1.37 | 1.04 | 1.79 | 26.91 | 4.24 | 44.22 |
| Granada | AMI | Theat | 1.05 | 1.00 | 1.11 | 5.18 | 0.24 | 9.88 |
| Granada | AMI | Tcold | 1.06 | 1.01 | 1.10 | 5.28 | 0.92 | 9.44 |
| Guadalajara | CVD | NO2 | 1.05 | 1.01 | 1.08 | 4.47 | 1.05 | 7.77 |
| Guipuzcoa | CVD | O3 | 1.03 | 1.00 | 1.05 | 2.49 | 0.35 | 4.57 |
| Guipuzcoa | CVD | Tcold | 1.09 | 1.05 | 1.13 | 8.13 | 4.98 | 11.19 |
| Guipuzcoa | ACVA | O3 | 1.07 | 1.03 | 1.12 | 6.61 | 2.52 | 10.53 |
| Guipuzcoa | IHD | Theat | 1.18 | 1.06 | 1.32 | 15.52 | 5.67 | 24.33 |
| Guipuzcoa | AMI | NO2 | 1.05 | 1.00 | 1.09 | 4.64 | 0.46 | 8.64 |
| Guipuzcoa | AMI | Tcold | 1.32 | 1.10 | 1.57 | 24.01 | 9.21 | 36.39 |
| Huelva | CVD | Tcold | 1.05 | 1.01 | 1.10 | 5.1 | 0.92 | 9.11 |
| Huelva | IHD | PM10 | 1.10 | 1.02 | 1.18 | 9.02 | 1.97 | 15.57 |
| Huelva | AMI | Tcold | 1.07 | 1.00 | 1.15 | 6.78 | 0.48 | 12.68 |
| Huesca | AMI | Tcold | 1.21 | 1.06 | 1.39 | 17.68 | 5.72 | 28.13 |
| Jaén | CVD | NO2 | 1.02 | 1.01 | 1.04 | 2.28 | 0.69 | 3.85 |
| Jaén | ACVA | NO2 | 1.04 | 1.00 | 1.07 | 3.52 | 0.04 | 6.88 |
| Jaén | ACVA | Theat | 1.16 | 1.05 | 1.29 | 13.87 | 4.65 | 22.2 |
| Jaén | IHD | Tcold | 1.87 | 1.07 | 3.28 | 46.6 | 6.58 | 69.47 |
| La Rioja | CVD | O3 | 1.05 | 1.00 | 1.10 | 4.98 | 0.45 | 9.31 |
| La Rioja | CVD | NO2 | 1.03 | 1.00 | 1.05 | 2.69 | 0.29 | 5.04 |
| La Rioja | CVD | Tcold | 1.14 | 1.07 | 1.21 | 12.46 | 6.91 | 17.67 |
| La Rioja | ACVA | Tcold | 1.15 | 1.00 | 1.32 | 13.13 | 0.31 | 24.31 |
| La Rioja | AMI | Tcold | 1.19 | 1.02 | 1.39 | 16.01 | 1.99 | 28.03 |
| Las Palmas | CVD | NO2 | 1.03 | 1.01 | 1.06 | 3.07 | 0.7 | 5.38 |
| Las Palmas | CVD | PM10 | 1.01 | 1.00 | 1.01 | 0.51 | 0.1 | 0.91 |
| Las Palmas | CVD | Theat | 1.05 | 1.00 | 1.10 | 5.09 | 0.49 | 9.48 |
| Las Palmas | ACVA | PM10 | 1.02 | 1.00 | 1.04 | 2.24 | 0.4 | 4.04 |
| Las Palmas | IHD | NO2 | 1.49 | 1.15 | 1.94 | 33.08 | 13.27 | 48.36 |
| Las Palmas | AMI | Tcold | 1.06 | 1.00 | 1.12 | 5.57 | 0.23 | 10.61 |
| Leon | CVD | NO2 | 1.03 | 1.01 | 1.05 | 2.82 | 1.09 | 4.53 |
| Leon | AMI | NO2 | 1.06 | 1.02 | 1.11 | 6.08 | 1.92 | 10.07 |
| Lérida | CVD | Tcold | 1.11 | 1.04 | 1.18 | 9.93 | 4.09 | 15.42 |
| Lérida | IHD | PM2.5 | 1.42 | 1.04 | 1.94 | 29.71 | 4.17 | 48.44 |
| Lugo | CVD | O3 | 1.01 | 1.00 | 1.03 | 1.44 | 0.32 | 2.54 |
| Lugo | CVD | PM2.5 | 1.06 | 1.03 | 1.09 | 5.49 | 2.45 | 8.43 |
| Lugo | CVD | Tcold | 1.24 | 1.02 | 1.49 | 19.2 | 2.43 | 33.09 |
| Lugo | ACVA | O3 | 1.03 | 1.00 | 1.05 | 2.52 | 0.33 | 4.66 |
| Lugo | ACVA | Tcold | 1.53 | 1.01 | 2.31 | 34.51 | 0.84 | 56.75 |
| Lugo | IHD | NO2 | 2.03 | 1.09 | 3.79 | 50.69 | 7.94 | 73.59 |
| Lugo | AMI | Tcold | 1.64 | 1.01 | 2.67 | 39.05 | 0.8 | 62.55 |
| Madrid | CVD | O3 | 1.08 | 1.06 | 1.11 | 7.67 | 5.41 | 9.88 |
| Madrid | CVD | Tcold | 1.04 | 1.03 | 1.05 | 3.87 | 3.13 | 4.6 |
| Madrid | ACVA | O3 | 1.16 | 1.10 | 1.23 | 14 | 8.75 | 18.95 |
| Madrid | AMI | O3 | 1.04 | 1.01 | 1.07 | 3.71 | 0.91 | 6.44 |
| Madrid | AMI | Theat | 1.05 | 1.00 | 1.10 | 4.68 | 0.06 | 9.09 |
| Malaga | CVD | Tcold | 1.18 | 1.05 | 1.32 | 15.35 | 5.15 | 24.45 |
| Malaga | ACVA | O3 | 1.02 | 1.00 | 1.03 | 1.51 | 0.05 | 2.94 |
| Malaga | ACVA | Tcold | 1.33 | 1.02 | 1.73 | 24.85 | 2.05 | 42.34 |
| Malaga | IHD | NO2 | 1.17 | 1.05 | 1.29 | 14.28 | 5.02 | 22.64 |
| Malaga | AMI | Tcold | 1.15 | 1.00 | 1.32 | 12.95 | 0.04 | 24.2 |
| Murcia | CVD | O3 | 1.03 | 1.02 | 1.04 | 2.99 | 1.75 | 4.21 |
| Murcia | CVD | Tcold | 1.05 | 1.03 | 1.07 | 4.71 | 2.89 | 6.5 |
| Murcia | ACVA | Tcold | 1.06 | 1.02 | 1.11 | 5.99 | 1.82 | 9.98 |
| Murcia | IHD | O3 | 1.22 | 1.09 | 1.38 | 18.33 | 8.06 | 27.46 |
| Murcia | AMI | PM10 | 1.03 | 1.01 | 1.05 | 2.67 | 1 | 4.31 |
| Murcia | AMI | Tcold | 1.08 | 1.03 | 1.13 | 7.2 | 2.54 | 11.64 |
| Navarre | CVD | NO2 | 1.02 | 1.00 | 1.03 | 1.82 | 0.26 | 3.36 |
| Navarre | CVD | Tcold | 1.10 | 1.02 | 1.18 | 8.73 | 1.55 | 15.38 |
| Navarre | ACVA | PM10 | 1.05 | 1.01 | 1.09 | 4.51 | 0.68 | 8.19 |
| Navarre | ACVA | Tcold | 1.17 | 1.01 | 1.37 | 14.73 | 0.76 | 26.74 |
| Ourense | CVD | Tcold | 1.02 | 1.00 | 1.03 | 1.77 | 0.21 | 3.31 |
| Ourense | ACVA | Tcold | 1.05 | 1.02 | 1.09 | 4.82 | 1.62 | 7.92 |
| Ourense | AMI | NO2 | 1.18 | 1.09 | 1.28 | 15.49 | 8.5 | 21.95 |
| Palencia | CVD | Tcold | 1.24 | 1.04 | 1.46 | 19.06 | 4.03 | 31.74 |
| Pontevedra | CVD | Tcold | 1.02 | 1.00 | 1.03 | 1.63 | 0.29 | 2.95 |
| Pontevedra | ACVA | PM10 | 1.04 | 1.02 | 1.07 | 4.25 | 2.28 | 6.18 |
| Pontevedra | ACVA | Tcold | 1.04 | 1.01 | 1.07 | 3.68 | 0.57 | 6.69 |
| Pontevedra | IHD | Tcold | 1.30 | 1.08 | 1.56 | 22.96 | 7.23 | 36.02 |
| Pontevedra | AMI | O3 | 1.02 | 1.01 | 1.04 | 2.11 | 0.68 | 3.52 |
| Pontevedra | AMI | Tcold | 1.08 | 1.03 | 1.12 | 7.08 | 3.33 | 10.68 |
| Salamanca | CVD | Tcold | 1.02 | 1.00 | 1.04 | 2.06 | 0.25 | 3.83 |
| Salamanca | ACVA | NO2 | 1.09 | 1.03 | 1.15 | 8.04 | 2.69 | 13.1 |
| Salamanca | AMI | Tcold | 1.07 | 1.02 | 1.12 | 6.14 | 1.62 | 10.46 |
| Segovia | CVD | Tcold | 1.05 | 1.02 | 1.09 | 5.05 | 1.56 | 8.42 |
| Segovia | ACVA | O3 | 1.05 | 1.02 | 1.07 | 4.42 | 1.83 | 6.95 |
| Segovia | ACVA | Tcold | 1.10 | 1.02 | 1.20 | 9.48 | 1.89 | 16.48 |
| Segovia | AMI | Tcold | 1.12 | 1.02 | 1.24 | 10.9 | 1.58 | 19.34 |
| Seville | CVD | Tcold | 1.05 | 1.02 | 1.08 | 4.99 | 2.12 | 7.77 |
| Seville | ACVA | Tcold | 1.09 | 1.01 | 1.18 | 8.51 | 1.35 | 15.15 |
| Seville | IHD | Tcold | 1.29 | 1.12 | 1.48 | 22.48 | 10.91 | 32.54 |
| Soria | CVD | O3 | 1.08 | 1.02 | 1.15 | 7.52 | 2 | 12.74 |
| Soria | CVD | Tcold | 1.13 | 1.01 | 1.27 | 11.63 | 1.03 | 21.11 |
| Soria | ACVA | O3 | 1.16 | 1.02 | 1.32 | 13.65 | 1.56 | 24.24 |
| Sta Cruz Tfe | CVD | Tcold | 1.12 | 1.03 | 1.22 | 10.6 | 2.63 | 17.92 |
| Sta Cruz Tfe | AMI | O3 | 1.03 | 1.01 | 1.06 | 3.02 | 0.63 | 5.36 |
| Tarragona | CVD | PM10 | 1.02 | 1.00 | 1.03 | 1.52 | 0.18 | 2.85 |
| Tarragona | CVD | Tcold | 1.05 | 1.02 | 1.08 | 4.59 | 1.88 | 7.22 |
| Tarragona | ACVA | Tcold | 1.08 | 1.01 | 1.16 | 7.65 | 1.24 | 13.64 |
| Teruel | CVD | Tcold | 1.16 | 1.05 | 1.29 | 13.99 | 4.6 | 22.46 |
| Teruel | ACVA | Theat | 1.43 | 1.08 | 1.88 | 29.84 | 7.68 | 46.68 |
| Teruel | ACVA | Tcold | 1.72 | 1.10 | 2.68 | 41.81 | 9.32 | 62.66 |
| Toledo | CVD | Tcold | 1.11 | 1.04 | 1.18 | 9.82 | 3.8 | 15.47 |
| Toledo | ACVA | NO2 | 1.08 | 1.03 | 1.13 | 7.57 | 3.11 | 11.81 |
| Toledo | ACVA | Tcold | 1.10 | 1.02 | 1.18 | 9.09 | 2.22 | 15.47 |
| Toledo | AMI | Tcold | 1.15 | 1.05 | 1.25 | 12.85 | 5.15 | 19.92 |
| Valencia | CVD | NO2 | 1.03 | 1.01 | 1.05 | 2.56 | 0.57 | 4.52 |
| Valencia | CVD | Tcold | 1.04 | 1.02 | 1.07 | 4.14 | 2.07 | 6.16 |
| Valencia | ACVA | Tcold | 1.03 | 1.00 | 1.05 | 2.7 | 0.23 | 5.11 |
| Valencia | IHD | NO2 | 1.16 | 1.06 | 1.26 | 13.42 | 5.8 | 20.43 |
| Valencia | IHD | Theat | 1.14 | 1.01 | 1.29 | 12.25 | 0.82 | 22.37 |
| Valencia | AMI | NO2 | 1.05 | 1.02 | 1.08 | 4.6 | 1.9 | 7.22 |
| Valencia | AMI | Tcold | 1.04 | 1.01 | 1.08 | 4.12 | 1.12 | 7.03 |
| Valladolid | CVD | Theat | 1.86 | 1.10 | 3.13 | 46.25 | 9.47 | 68.09 |
| Valladolid | CVD | Tcold | 1.05 | 1.02 | 1.08 | 4.59 | 1.99 | 7.12 |
| Valladolid | ACVA | Theat | 4.25 | 1.77 | 10.19 | 76.48 | 43.62 | 90.19 |
| Valladolid | ACVA | Tcold | 1.11 | 1.04 | 1.18 | 9.61 | 3.6 | 15.25 |
| Valladolid | AMI | Tcold | 1.10 | 1.02 | 1.18 | 8.83 | 2.07 | 15.12 |
| Vizcaya | CVD | PM2.5 | 1.02 | 1.00 | 1.03 | 1.79 | 0.48 | 3.09 |
| Vizcaya | CVD | Tcold | 1.05 | 1.02 | 1.08 | 4.39 | 1.69 | 7.01 |
| Vizcaya | ACVA | NO2 | 1.03 | 1.01 | 1.06 | 3.13 | 0.64 | 5.56 |
| Vizcaya | IHD | PM10 | 1.17 | 1.01 | 1.36 | 14.51 | 0.53 | 26.54 |
| Zamora | CVD | NO2 | 1.10 | 1.04 | 1.16 | 9.12 | 4.11 | 13.86 |
| Zamora | CVD | Tcold | 1.05 | 1.02 | 1.08 | 4.93 | 2.08 | 7.7 |
| Zamora | ACVA | Tcold | 1.11 | 1.04 | 1.18 | 9.76 | 3.64 | 15.5 |
| Zamora | AMI | NO2 | 1.29 | 1.07 | 1.55 | 22.22 | 6.27 | 35.45 |
| Zamora | AMI | Tcold | 1.09 | 1.00 | 1.19 | 8.46 | 0.15 | 16.08 |
| Zaragoza | ACVA | Tcold | 1.06 | 1.01 | 1.12 | 5.83 | 0.87 | 10.55 |
| Zaragoza | IHD | PM10 | 1.17 | 1.01 | 1.36 | 14.44 | 0.77 | 26.23 |
| Zaragoza | AMI | Theat | 1.13 | 1.02 | 1.26 | 11.77 | 2.15 | 20.44 |
